# Quantitative Structure–Activity Relationship in the Series of 5-Ethyluridine, N2-Guanine, and 6-Oxopurine Derivatives with Pronounced Anti-Herpetic Activity

**DOI:** 10.3390/molecules28237715

**Published:** 2023-11-22

**Authors:** Veronika Khairullina, Yuliya Martynova

**Affiliations:** Institute of Chemistry and Defence in Emergency Situations, Ufa University of Science and Technology, 50076 Ufa, Russia; martynovayuz@uust.ru

**Keywords:** inhibitors of herpes simplex virus thymidine kinase, HSV-1, HSV-2, QSAR models, GUSAR 2019 program, QNA descriptors, MNA descriptors, structure–activity relationships

## Abstract

A quantitative analysis of the relationship between the structure and inhibitory activity against the herpes simplex virus thymidine kinase (HSV-TK) was performed for the series of 5-ethyluridine, N2-guanine, and 6-oxopurines derivatives with pronounced anti-herpetic activity (IC_50_ = 0.09 ÷ 160,000 μmol/L) using the GUSAR 2019 software. On the basis of the MNA and QNA descriptors and whole-molecule descriptors using the self-consistent regression, 12 statistically significant consensus models for predicting numerical pIC_50_ values were constructed. These models demonstrated high predictive accuracy for the training and test sets. Molecular fragments of HSV-1 and HSV-2 TK inhibitors that enhance or diminish the anti-herpetic activity are considered. Virtual screening of the ChEMBL database using the developed QSAR models revealed 42 new effective HSV-1 and HSV-2 TK inhibitors. These compounds are promising for further research. The obtained data open up new opportunities for developing novel effective inhibitors of TK.

## 1. Introduction

Herpes virus infections induced by viruses of the Herpesviridae family are among the most widespread human diseases. Antibodies to various herpes viruses are identified in about 95% of the world’s population. Eight types of Herpesviridae viruses cause herpesvirus infections [1,2,3]. Herpes simplex viruses of the first and second type (HSV-1 and HSV-2) are the most common [4]. HSV-1 usually affects the upper body (mouth, eyes, and brain), whereas HSV-2 relates to genital infections [5]. These viruses are usually latent. However, when immunity is reduced, they are activated, which, in turn, provokes diseases, such as oral herpes, genital herpes, keratitis, conjunctivitis, herpes zoster, etc. It is reported that herpes virus infections induced by HSV-1 and HSV-2 (and other types) increase the possibility to be infected with the human immunodeficiency virus (HIV) and are almost always diagnosed in patients with an HIV infection, which complicates the course of this disease. There is evidence that HSV-1 can participate in the development of multiple sclerosis [6] and lead to male infertility [7].

Currently, there are three classes of drugs in active medical practice for the treatment of infectious diseases caused by different types of herpes viruses, including HSV-1 and HSV-2: (1) acyclic guanosine analogues; (2) acyclic nucleotide analogues; and (3) pyrophosphate analogues (foscarnet) [8].

Acyclovir is known to be an effective inhibitor of viral thymidine kinase (TK). This drug is the gold standard for the prevention and treatment of infections caused by HSV-1 and HSV-2, as this drug combines a pronounced clinical effect and low toxicity [9]. However, a significant disadvantage of acyclovir is its low oral bioavailability, poor solubility, and short blood circulation time. An increase in the therapeutic dose of this drug is undesirable with long-term use, as it leads to an increase in its toxicity. Another disadvantage of acyclovir is related to drug resistance development. This problem is not significant for patients with good immunity, since the incidence of acyclovir-resistant herpes simplex virus strains among them is ~0.5% of cases. However, among patients with immunodeficiency conditions, it exceeds 30% of cases. In 95% of cases, acyclovir resistance is due to mutations in the thymidine kinase and DNA polymerase genes, which are related to the mechanism of action of this drug [10,11,12,13,14,15]. In addition to acyclovir, the phenomenon of drug resistance was observed for pentacyclovir and its analogues used as HSV-1- and HSV-2-replication inhibitors. The analogues of adenine, adefovir, and tenofovir have found the greatest application in clinical practice among the phosphonate derivatives of guanosine. These drugs are included in therapy to suppress HSV-1 strains resistant to acyclovir (and its analogues) and some similar cases with the deficiency of viral thymidine kinase. The same drugs are used in the treatment of hepatitis B and HIV. However, these drugs have a pronounced nephro- and hepatotoxic effect. Foscarnet, a covalent inhibitor of DNA polymerase, has not been widely used in clinical practice due to the rather high toxicity and the lack of selectivity. The above-mentioned issues demonstrate the necessity for the search for new anti-herpetic drugs [8].

Additional promising strategies against the HSV-induced herpes infections deal with the development of inhibitors of other enzymes (helicase-primase or ribonucleotide reductase) and inhibitors of the adhesion/penetration of the virus into the cell. Currently, inhibitors of the mentioned enzymes are at various stages of preclinical and clinical trials and out of medical practice.

Thymidine kinase inhibitors occupy a special place in the development of new-generation antiviral drugs. It should be noted that this enzyme plays a key role in the thymidine metabolism both in healthy and virus-infected cells. In healthy cells, this intracellular enzyme catalyzes the conversion of thymidine to thymidine monophosphate (TMP) in the presence of adenosine triphosphate (ATP). Viral thymidine kinase differs from the thymidine kinase of the host cell in its much greater substrate specificity and it is able to catalyze the phosphorylation of thymidine, pyrimidines, and purines. Subsequently, in both healthy and virus-infected cells, the resulting monophosphates of pyrimidines and purines are converted into the corresponding bi- and triphosphates. Triphosphates are then incorporated into deoxyribonucleic acid. Thus, viral thymidine kinase inhibitors cannot be simultaneously used with preparations containing acyclovir and its analogues as an active component [5,8].

DNA polymerase inhibitors prevent the replication of the virus after reactivation. In contrast, thymidine kinase inhibitors are aimed at preventing reactivation by lengthening the latent period of the virus. However, when developing antiviral drugs based on thymidine kinase, one should bear in mind that viral thymidine kinase is not a key target in the replication of herpes virus in rapidly dividing cells where the amount of thymidine triphosphate is sufficient for the synthesis of viral DNA due to cellular metabolism. However, this enzyme plays a key role in non-proliferating (non-dividing) nerve cells, in which the synthesis of cellular DNA occurs at a low level (if at all). In this case, the inhibition of viral thymidine kinase leads to the growth of damaged and, therefore, non-viable cells in primary neuronal cultures.

In addition to a potential target in the fight against viral infections, thymidine kinase (TK) is considered a tumor marker, which is used to diagnose and monitor the increased proliferation of tumor cells. It is known that tumor cells have an increased concentration of TK due to their high-intensity division and growth.

Thus, the search for efficient TK inhibitors, including TK of the human herpes viruses HSV-1 and HSV-2, can be considered one of the promising medical treatment options for herpetic infections and cancer diseases of various origins [16,17]. However, the rational search for new drugs without involving virtual screening methods is impractical both from an economic point of view and because of the high time costs [18,19,20,21,22].

In this regard, scientists use various approaches for computer-aided drug design (CADD) to search for hit compounds at the initial stage of development of new potential drugs [23]. There are known drugs that have been developed using this approach, such as tirofiban [24], zanamivir [25], boceprevir [26], saquinavir [27], captopril [28,29], and aliskiren [30]. CADD approaches are classified into structure-based and ligand-based methods. In the first category of methods, computational drug design is carried out by studying the interactions between ligands and target molecules. Accordingly, the aim of this variant of CADD is to optimize the binding structure of the ligand under study and the corresponding receptor in a three-dimensional form. Virtual protein–ligand complexes are modeled using pharmacophore search, molecular docking, and molecular dynamics methods. The most obvious disadvantage of structure-based approaches in CADD is the requirement of correct information on the receptor structure and the high time and computational costs [31].

In the alternative category of ligand-based CADD approaches, the leading factors contributing to biological activity are the physicochemical, electronic, and conformational features of the ligands. The key advantage of the latter strategy over the former one is mainly that in the latter case, knowledge of the spatial structure and amino acid composition of the target is not required for the design of potential drugs [23,31].

Quantitative structure–activity relationship (QSAR) is a valuable method in CADD which aims to build statistically significant mathematical models for predicting different biological activity parameters (pIC_50_, pLD_50_, pK_i_, etc.) based on different physicochemical, electronic, and structural characteristics of organic compounds [32,33]. In terms of dimensionality, the type of QSAR models depends on the descriptors used, ranging from 0D-QSAR to 7D-QSAR [34]. Several descriptors (e.g., atomic properties, number of fragments, and topological descriptors) make up the 0D to 2D-QSAR components. Modeling using 3D-QSAR methods requires the inclusion of 3D descriptors giving an additional dimension in spatial coordinates [35,36]. Additional aspects of 3D-QSAR models require the use of multidimensional molecular descriptors based on conformational flexibility, induced fit, solvation function, and target-based receptor models. These supplements generate multidimensional QSAR (i.e., 4D to 7D-QSARs) [32]. A factor complicating the practical use of 3D-7D QSAR methods is the required knowledge of the bioactive conformation of the ligands that are structural analogues of the compounds being modeled [37,38,39]. Taking into account of all of the above factors in terms of time and computational cost can, in some cases, be far superior to the first category of structure-based CADD methods. In this regard, today there is a growing interest in the use of 2D-QSAR models against the background of a relatively smaller number of studies using multivariate QSAR approaches despite the high predictive power, logical validity, and objectivity of the latter.

The GUSAR program, developed at the V.N. Orekhovich Institute of Biomedical Chemistry of the Russian Academy of Medical Sciences, is modern software for the construction of quantitative and classification models (QSAR and SAR models) and the prediction of various types of biological activity, as well as other properties of organic compounds based on a 2D approach (structural formulae of organic compounds) [20]. In this software, the chemical structure is described in terms of descriptors called quantitative neighborhoods of atoms (QNAs) and multilevel neighborhoods of atoms (MNAs) developed at the same institute [40,41,42]. The functioning algorithm of this software is based on the method of self-consistent regression previously developed by the same team with the inclusion of additional estimates of the quality of prediction of the target property (based on the method of nearest neighbors and artificial neural network with a radial basis function) and construction of a consensus of the set of models [20]. It is reported that GUSAR is not inferior to other methods (CoMFA, CoMSIA, HQSAR, etc.) used to build QSAR/QSPR models in terms of accuracy and predictive ability [43,44]. As a result, the software can be successfully applied to a variety of QSAR/QSPR tasks [45,46,47,48,49,50,51,52,53,54,55,56,57,58]. In particular, the GUSAR software has been used for more than a decade to model various types of biological activity and toxicity of organic compounds [40,41,42,43,44,45,46,47,48,49]. In addition, the successful application of this software has been demonstrated for the QSPR modeling of several physicochemical properties of organic compounds, including the n-octanol–water partition coefficient (logP) [45], boiling and melting points, density, thermal conductivity, viscosity, surface tension, water solubility, and gas pressure [40].

Additionally, our earlier publications demonstrated the successful application of this software for QSPR modeling of antioxidants under conditions of the liquid-phase radical-chain oxidation of organic substrates [59,60,61,62,63].

This software has been used for more than a dozen years for modeling different types of biological activity. It was shown by the developers and other researchers, including our research team, that GUSAR software can be successfully applied to multiple QSAR/QSPR problems [50,51,52,53,54,55,56,57,58,59,60,61,62,63].

**Figure 1:**
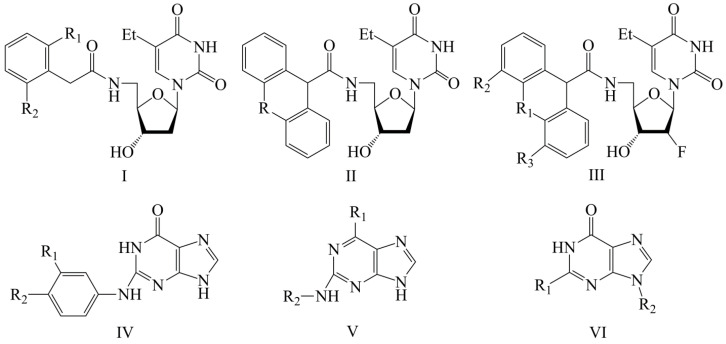
General structural formulas of simulated inhibitors of HSV-1 and HSV-2 thymidine kinases based on a series of 5′-amino-2′,5′-dideoxy-5-ethyluridine (I–III), N2-phenylguanine (IV), and 2-phenylamino-6-oxopurine carboxamide derivatives (V,VI).

In this work, we used GUSAR 2019 software to study the quantitative structure–activity relationship for inhibitors of HSV-1 and HSV-2 viral thymidine kinase using the series of 5′-amino-2′,5′-dideoxy-5-ethyluridine (I–III), N2-phenylguanine (IV), and 2-phenylamino-6-oxopurine carboxamide derivatives (V–VI, Figure 1) and developed coupled and statistically significant QSAR models for screening virtual libraries and databases.

## 2. Results

Using the consensus approach implemented in the GUSAR 2019 program, we have studied the quantitative relationship between the structure and the efficiency of inhibition of HSV-1 and HSV-2 TK with 5-ethyluridine, N2-guanine, and 6-oxopurine derivatives with general structural formulas I–VI (Figure 1). These compounds made up the training sets TrS1–TrS4. Depending on the type of descriptors used in the calculations (MNA and/or QNA), three QSAR consensus models have been obtained for each of the training sets. In total, we have built 12 QSAR consensus models for predicting pIC_50_ values for HSV-1 and HSV-2 TK inhibitors that included from 20 to 360 partial regression models. The pIC_50_ values for inhibitors included in TrS1–TrS4 derived from these QSAR consensus models M1–M12 were compared with the experimental values of pIC_50_ (see Tables S2–S5 in Supplementary Materials).

The regression models were not explicitly displayed, as a clear physical interpretation of the descriptors was absent. Hence, we could not determine the descriptors making the largest/the smallest contributions to the simulated activity [64,65]. However, this was beyond the scope of this study. Our goal was to solve two problems:

(1) to show that the ideology of descriptor formation and selection implemented in the GUSAR 2019 software is applicable for modeling potential inhibitors of HSV-1 and HSV-2 TK enzymes in the series of 5-ethyluridine, N2-guanine, and 6-oxopurine derivatives;
(2) to develop statistically significant QSAR models suitable for the virtual screening of HSV TK inhibitors.

For the internal validation of the QSAR models M1–M12 over the TrS1–TrS4 structures, we used a cross validation procedure with a 20-fold randomized exclusion of 20% of the compounds. Here, the averaged values of determination coefficients $\bar{R^{2}}$ and $\bar{Q^{2}}$ for the inhibitors of all training sets were similar (Table 1); the difference between these two indicators (Δ = $\bar{R^{2}}$ − $\bar{Q^{2}}$) did not exceed 0.1. This assessment indicates the stability of the constructed consensus models.

This is exemplified in Table 1, Table 2 and Table 3, which present the numerical values of the statistical criteria estimated by comparing the experimental and predicted pIC_50_ values calculated using models M1–M12 with 95% of the data included in the corresponding training set. Full information about all of these criteria using the twelve developed QSAR models, which enables an objective evaluation of the descriptive and predictive ability of the models, taking into account 95% and 100% of the data included in the training and test sets, respectively, is given in the Supplementary Materials (Tables S2–S5).

The data of Table 1, Table 2 and Table 3 provide the conclusion that all constructed QSAR models had high descriptive ability. However, the data presented in Table 1, Table 2 and Table 3 clearly demonstrate the discrepancy between the numerical values of determination coefficients (R^2^) found while evaluating the descriptive ability of models M1–M12 in the GUSAR 2019 and XternalValidationPlus 1.2 software, due to different ideologies underlying the calculations.

It should be taken into account that in the GUSAR 2019 software, the target parameter (in our case, pIC_50_) for each chemical structure included in the training or test set is predicted as a result of averaging the numerical values of this parameter calculated using each of the particular models included in a single consensus model. The final statistical parameters are calculated in a similar way.

For example, when predicting pIC_50_ values for any compound from the training set TrS1 using the consensus model M1, we get a set of 20 predicted pIC_50 pred_ values and 20 sets of different internal validation criteria: R^2^, Q^2^, F, and SD. Further, all the same data are averaged, which is displayed as the final results.

Meanwhile, in the XternalValidationPlus 1.2 program, the calculation of statistical parameters for assessing the descriptive and predictive ability of QSAR models is based on comparing the experimental pIC_50_ data with the average values previously predicted using the GUSAR 2019 software. This procedure is performed twice without averaging the final results [66]:

(1) for the full dataset in each training and test set (100% of data);
(2) for 95% of the data in each training and test set (95% of the data).

In general, a comparison of the data given in Table 1, Table 2 and Table 3 demonstrates that the SCR method of GUSAR 2019 for selecting significant descriptors produces stable regression dependences with acceptable statistical characteristics (R^2^_TrS_ > 0.6 and Q^2^_TrS_ > 0.5) for simulated HSV-1 and HSV-2 TK inhibitors, regardless of the selected types of descriptors.

The different determination criteria of the descriptive ability of models M1–M12 are similar irrespective of the amount of data in the sets (95 or 100%) and tend to be 1 (Table 2 and Table 3). The MAE error values do not exceed 15% of the ΔpIC_50_ range of the inhibitory activity of the TrS1–TrS4 structures. The parameter ΔR^2^_m_ is in all cases is much lower than 0.2 and does not exceed 0.048. All of these data indicate the rather high simulability of the target properties using the selected algorithms for a calculation of descriptors and construction of regression equations [67] implemented in the GUSAR 2019 software.

An external validation of the M1–M3 and M7–M9 QSAR models was performed by predicting the pIC_50_ for HSV-1 TK inhibitors using test sets TS1 and TS3. The validity of the models M4–M6 and M10–M12, meant for the prediction of the pIC_5_ for HSV-2 TK inhibitors, was evaluated in relation to test sets TS2 and TS4. All estimates of the predictive ability of the M1–M12 models were based on three criteria:

(1) numerical values of various coefficients of determination based on R^2^ (R^2^, R^2^_0_, Q^2^_F1_, Q^2^_F2_, CCC);
(2) numerical values of the MAE prediction error;
(3) the scatter range of activity prediction data taking into account MAE in the mσ (or mSD) range: MAE + 3·SD. All of these parameters were computed using the XternalValidationPlus 1.2 program. In addition, this program was used to trace the systematic error that can arise in QSAR modeling.

**Figure 2:**
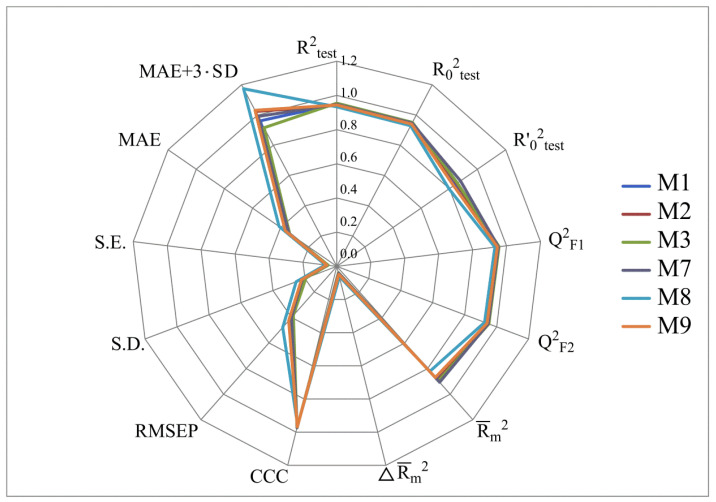
Distribution of statistical characteristics of QSAR models derived from predicted pIC_50_ values for the structures of the external test set TS1.

**Figure 3:**
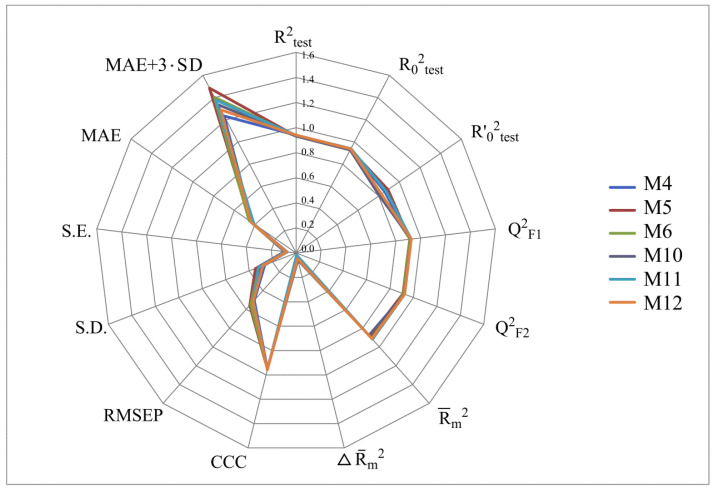
Distribution of statistical characteristics of QSAR models derived from the predicted pIC_50_ values for the structures of the external test set TS2.

**Figure 4:**
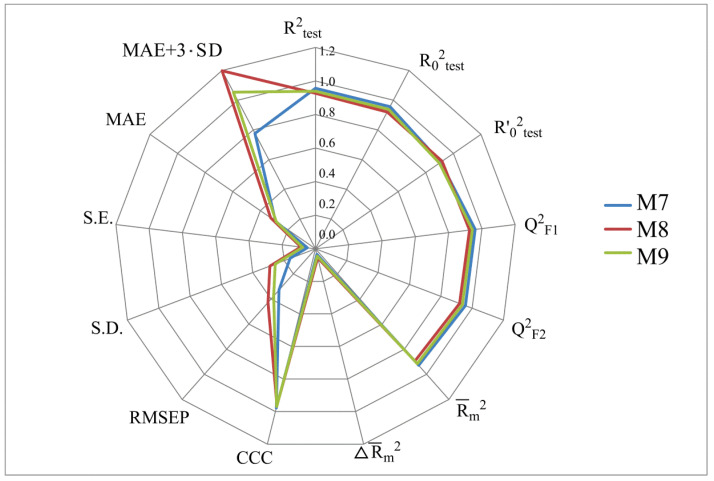
Distribution of statistical characteristics of QSAR models derived from the predicted pIC_50_ values for the structures of the external test set TS3.

**Figure 5:**
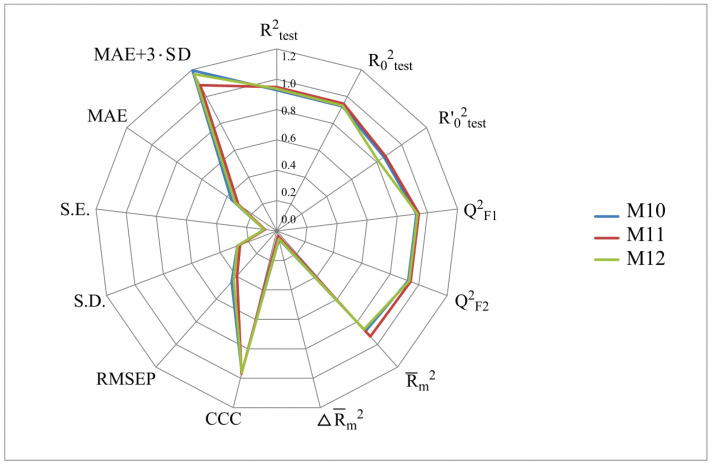
Distribution of statistical characteristics of QSAR models derived from the predicted pIC_50_ values for the structures of the external test set TS4.

Figure 2, Figure 3, Figure 4 and Figure 5 show the distribution of different determination coefficients and prediction errors for pIC_50_ values for 95% of the HSV inhibitors from test sets TS1–TS4 calculated using the XternalValidationPlus 1.2 program. The complete set of all statistical parameters obtained from a comparison of experimental and predicted pIC_50_ values for the TS1–TS4 structures determined based on models M1–M12 is given in Tables S2–S5 (Supplementary Materials).

The more stringent criterion $\bar{R_{m}^{2}}$ is relatively high for the external validation of models M1–M12 using the full size of TS1–TS4, being in the range of 0.8273–0.8859 and 0.7587–0.8683 for HSV-1 and HSV-2 inhibitors, respectively. After removing 5% of outliers from TS1–TS4, the ranges of $\bar{R_{m}^{2}}$ become 0.8207–0.9294 and 0.8664–0.9294 for HSV-1 and HSV-2 inhibitors, respectively (Figure 2, Figure 3, Figure 4 and Figure 5, Tables S2–S5 in Supplementary Materials). The Δ$\bar{R_{m}^{2}}$ criterion, proposed by the same authors as an additional parameter for assessing the predictive ability for the external validation of regression models, did not exceed 0.09 in any of the cases. This also indicates the rather high predictive ability of QSAR models M1–M12 (Figure 2, Figure 3, Figure 4 and Figure 5, Tables S6–S13 in Supplementary Materials).

Based on a comparison of different determination coefficients obtained during the external validation of models M1–M12, we have found that the parameter pIC_50_ for 5-ethyluridine, N2-guanine, and 6-oxopurine derivatives with respect to HSV-1 is modeled with higher accuracy than that for the same compounds against HSV-2.

As we noted above, an analysis of different types of determination coefficients is faced with the following contradictory situation: the R^2^ and R^2^_0_ values for the activity of 5-ethyluridine, N2-guanine, and 6-oxopurine derivatives against HSV-1 are equal to or less than Q^2^_F1_ and Q^2^_F2_. This means that the constructed models M1–M12 predict the activities of TS1–TS4 compounds better than the activities of the training set structures. Note that in practice, the situation is usually opposite. This fact was repeatedly noted by other researchers [68,69,70,71]. Thus, the use of the metrics based on R^2^ and Q^2^ alone for assessing the predictive ability of QSAR models seems to be insufficient.

According to these two criteria, the predictive ability of models M1–M3, M7, and M9 has been classified as high for both test sets TS1 and TS3. Since the MAE + 3·SD criterion has been at the boundary of the allowable threshold value equal to 1.1735, the predictive ability of model M8 for 95% of the data of test set TS1 is moderate. The MAE and MAE + 3·SD values in the case of sets TS2 and TS4 do not exceed 0.6250 and 1.5625, respectively. As a result, the predictive ability of model M4 in the sets TS2 and TS4 has also been rated as high. At the same time, considering these two threshold values, the predictive abilities of M5–M12 can be estimated as satisfactory for set TS2 and as high for set TS4.

A comparative analysis of the statistical characteristics and prediction errors of pIC_50_ indicate that all constructed models have rather high descriptive and predictive ability. However, to solve the problem of searching for new potential inhibitors of HSV-1 and HSV-2 TK enzymes among the title compounds, it is most preferable to use the consensus models M3 and M6 because they include 100 particular regression models and each of them is based on the maximum set of structures and descriptors.

In this regard, we have applied the consensus models M3 and M6 to virtual screening through the CHEMBL database for new HSV TK inhibitors among various lead compounds and active drug components of different pharmacological profiles. Unlike traditional methods of QSAR modeling (multilinear regression (MLR), partial least squares (PLS) method, etc.), the GUSAR software does not specify clear threshold criteria regarding the Tanimoto coefficient, which would limit the search for new potential biologically active substances in virtual databases. However, adhering to concepts of the classical school, we limited the scope of the search for new potential inhibitors of HSV-1 TK and HSV-2 TK in the ChEMBL database by the degree of similarity of at least 70% with respect to the reference compounds.

The virtual screening involved 400 5-ethyluridine, N2-guanine, and 6-oxopurine derivatives with pronounced antitumor and antibacterial properties and no antiviral properties. However, only 192 lead compounds and known pharmaceuticals fitted in the range of applicability of consensus models M3 and M6. For 155 structures of these, the predicted IC_50_ values were <1 μmol/L. The most promising hit compounds are presented in Table 4. The complete list of the structures of the potential HSV TK inhibitors predicted using consensus models M3 and M6 is given in Table S14 in the Supplementary Materials. We assume that in living systems, these compounds should behave as multi-target drugs. They are promising for further detailed studies.

**Figure 6:**
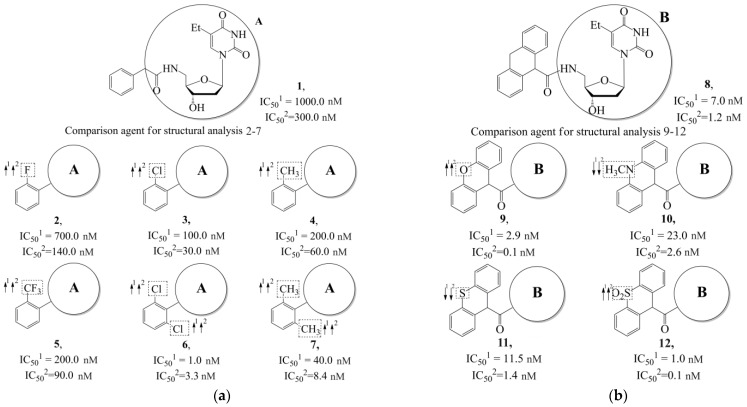
Effect of acyclic substituents on the activity of herpes virus inhibitors with general formulas I and II with the chemical group contributions to the activity; superscripts 1 and 2 refer to the activities against HSV-1 TK and HSV-2 TK, respectively. Dotted lines highlight the substituents. The up and down arrows indicate the positive or negative effect of the selected group. A and B denote fragments that remained unchanged during structural analysis.

**Figure 7:**
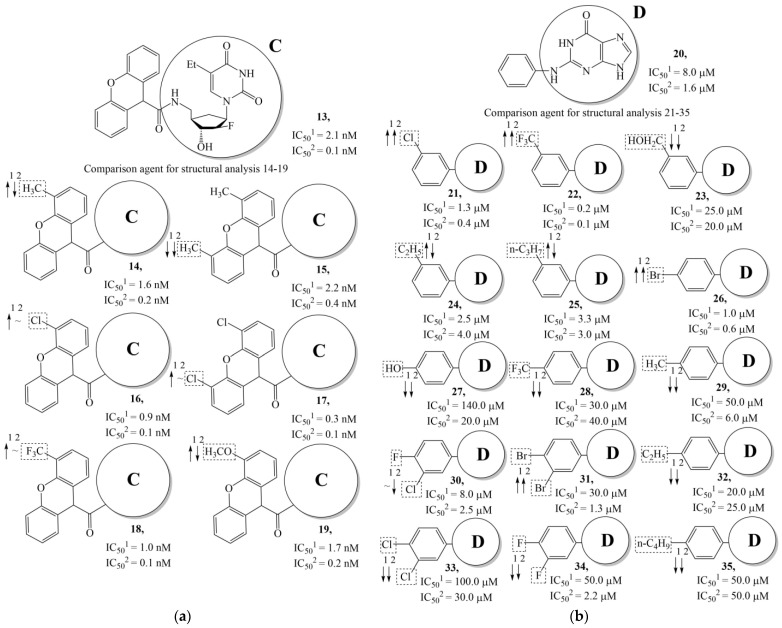
Effect of acyclic substituents on the activity of herpes virus inhibitors with general formulas III–IV with the chemical group contributions to the activity; the superscripts 1 and 2 refer to activities against HSV-1 TK and HSV-2 TK, respectively. The dotted lines highlight the substituents. The up and down arrows indicate the positive or negative effect of the selected group. C and D denote fragments that remained unchanged during structural analysis.

**Figure 8:**
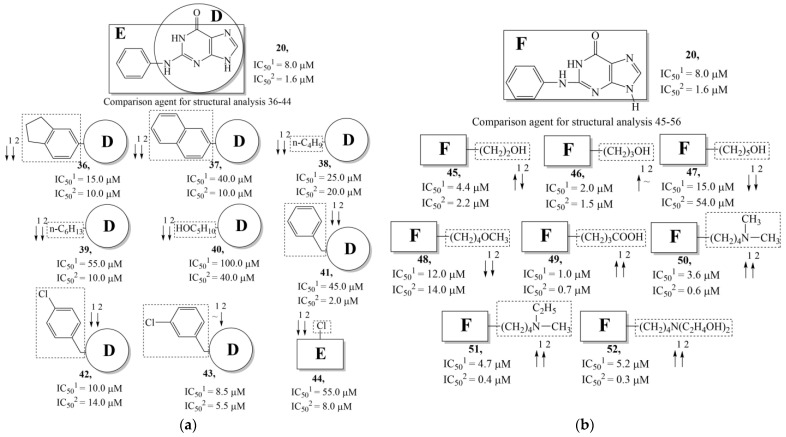
Effect of acyclic substituents on the activity of herpes virus inhibitors with general formulas V–VI with the chemical group contributions to the activity, where superscripts 1 and 2 refer to activities against HSV-1 TK and HSV-2 TK, respectively. Dotted lines highlight substituents. The up and down arrows indicate the positive or negative effect of the selected group. D, E and F denote fragments that remained unchanged during structural analysis.

Additionally, using the GUSAR 2019 program, we carried out a structural analysis of TK inhibitors. Since for 42 compounds presented in Table 4, there were no experimental data on the inhibitory activity against human herpes viruses HSV-1 and HSV-2, these compounds were not included in the structural analysis. We used the consensus model M3, as it provides more objective and accurate results due to the maximum number of modeled structures and involvement of all types of descriptors implemented in GUSAR 2019 [20,21,22]. However, it should be noted that these compounds have been extensively studied for their inhibitory activity against HPV in previous biological experiments [17,72,73]. Therefore, here, we will briefly discuss this issue. Figure 6, Figure 7 and Figure 8 show the analysis of the contribution of different functional groups to the activity of inhibitors of HSV-1 and HSV-2 thymidine kinase with general structural formulas I–VI. For compounds with the general structural formula I, it was experimentally shown that the replacement of a hydrogen atom in the R_1_ position of the benzene ring (**1**) increases the inhibitory activity, irrespective of the nature of the acyclic substituent. The results of a structural analysis of the same compounds obtained using the GUSAR 2019 program lead to a similar conclusion. This enhancement is manifested for compounds **2**–**7** containing fluoro (**2**), chloro (**3**), methyl (**4**), and trifluoromethyl (**5**) substituents in the ortho-positions (Figure 6a). 

In compounds with the general formula II, replacement of the dihydroxanthene moiety (**8**) with a xanthene (**9**) or thioxanthene dioxide (**12**) moiety somewhat increases the activity of the TK inhibitors of HSV-1 and HSV-2. At the same time, replacement by dibenzosuberene, anthracene, or NMe-acridine (**10**) has an adverse effect on both target properties. Note that the first two of these groups induce a pronounced decrease in the inhibitory activity, while the third replacement has only a moderate effect. The replacement of the dihydroxanthene moiety in **8** with a thioxanthene moiety (**11**) decreases the inhibitory activity against HSV-1 TK by a factor of 1.5 and has almost no effect on the inhibitory activity against HSV-2 TK (Figure 6b).

In compounds with the general structural formula III containing an oxygen atom in position R_1_ (i.e., xanthene ring, **13**) (Figure 7a), the replacement of the hydrogen atom in position R_2_ with a methyl group (**14**) increases the inhibitory activity against HSV-1 TK and impairs the activity of TK inhibitors against HSV-2. However, the effect is not clearly pronounced in both cases. The introduction of a second methyl group into position R_3_ (**15**) of the xanthene ring decreases both target properties. The alternative replacement of the hydrogen atom at position R_2_ by a chlorine atom (**16**) increases the activity of TK inhibitors for HSV-1 almost 2-fold, but barely affects the inhibitory activity against HSV-2 TK. The additional incorporation of a second chlorine atom at position R_3_ (**17**) is favorable for the activity against HSV-1 TK and almost does not influence the activity against HSV-2 TK. The replacement of the hydrogen atom in position R_2_ with a trifluoromethyl group (**18**) and unsubstituted phenyl increases the TK inhibitory activity against HSV-1 and has almost no effect on this activity against HSV-2. Meanwhile, the modification of position R_2_ by introducing a methoxy group (**19**) increases the activity of HSV-1 TK inhibitors and decreases the activity of HSV-2 TK inhibitors. However, the changes caused by a hydrogen atom replacement with the above substituents are moderate.

In compounds with the general structural formula IV (Figure 7b), the replacement of the hydrogen atom (**20**) in the meta-position by chlorine (**21**) or a trifluoromethyl group (**22**) increases the activity of both TK isoforms quite significantly. Modification of the meta-position in the benzene ring with a hydroxymethyl group (**23**) negatively affects both target properties, and the adverse effect is high. At the same time, the alternative replacement of the hydrogen atom with ethyl (**24**) or n-propyl (**25**) increases the activity of TK inhibition of HSV-1 and decreases the activity of TK inhibition of HSV-2.

The replacement of hydrogen in the para-position with a bromine atom (**26**) favorably affects both target properties. In contrast, the alternative replacement of hydrogen with methyl (**29**), ethyl (**32**), n-butyl (**35**), trifluoromethyl (**28**), or hydroxyl (**27**) markedly decreases the inhibitory activity of compounds with the general structural formula IV against both TK isoforms (Figure 7b).

The simultaneous substitution of hydrogen atoms in the meta- and para-positions of the benzene ring by a bromine atom (**31**) considerably increases the efficiency of inhibitors of HSV-1 TK and almost does not affect the efficiency against HSV-2 TK. However, if we consider this substitution as sequential, the introduction of the second bromine atom in the meta-position of the benzene ring decreases the activity of both TKs compared to the modification of only the para-position by this substituent. The inclusion of fluorine and chlorine atoms in the para- and meta-positions (**30**) of the benzene ring, respectively, does not affect the inhibitory efficiency against HSV-1 and markedly decreases that against HSV-2. Similar modifications of para- and meta-positions based on the inclusion of two chlorine (**33**) or fluorine atoms (**34**) significantly decrease both target properties (Figure 7b).

The replacement of benzene (**20**) with a 2,3-dihydro-1H-indene (**36**) or naphthalene (**37**) ring and with a number of acyclic groups, including n-butyl (**38**), n-hexyl (**39**), and 1-hydroxypentyl (**40**), in compounds with general structural formula V has the same effect (Figure 8a).

In addition, in compounds with the general structural formula V, replacement of the benzene ring (**20**) in position R_2_ with a benzyl moiety (**41**) markedly reduces the efficiency of inhibition of HSV-1 TK and has almost no effect on the activity of HSV-2 TK. At the same time, structural analogues of benzyl containing a chlorine atom in the meta- (**43**) or para-position (**42**) have the opposite effect, which is also markedly pronounced. The replacement of the oxo group (hydroxyl group, if we consider the alternative resonance structure) with a chlorine atom (**44**) and a hydroxyl group significantly reduces the inhibitory activity against both TK isoforms (Figure 8a).

In compounds of general formula VI, the introduction of hydroxyalkyl, aminoalkyl, or carboxyalkyl substituents in position 9 (position R_2_) of the purine ring, except for 2-hydroxyethyl (**45**) and 3-hydroxypentyl (**46**), increases both target properties. The introduction of 4-(piperidinyl)butyl and its derivatives containing a benzene moiety and acyclic substituents at positions 2, 3, and 4 of the pyridine ring has a similar effect. The only exceptions in the latter case are the two oxopurine derivatives with the general structural formula VI containing 4-(4-hydroxypyridyl)butyl and 4-(1,4′-bipyridine)butyl at position R_1_. However, these two moieties have a negative effect only for the inhibition of the TK activity of HSV-1. The activities of HSV-2 TK inhibitors are not affected by these two modifications. The modification of the R_2_ position in the oxopurine ring by replacing the hydrogen atom with 4-(decahydroquinolyl)butyl or 4-(1,2,3,4-tetrahydroquinolyl)butyl makes a positive contribution to both target properties (Figure 8b).

In oxopurine derivatives with the general formula VI containing a 4-hydroxyl group at position R_2_, the replacement of the phenylamine moiety at position R_1_ with a primary amino group or with a methylamine moiety significantly decreases the inhibitory activity against both TKs. Meanwhile, the introduction of a 2-phenoxyl or 2-phenylthiol moiety instead of 2-phenylaminyl moiety promotes the activity of inhibitors of HSV-1 TK, but negatively affects the inhibition efficiency of HSV-2 TK.

Overall, the comparison of experimental and calculated data indicates that the results of the structural analysis performed in GUSAR-2019 were 80% consistent with the results of previous biological studies.

The discrepancies in predicted estimates of the influence of structural descriptors on the target activities are observed only for the simulated structures containing bulky cyclic moieties, such as dibenzosuberene, NMe-acridine, thioxanthene dioxide, and their structural analogues. The mismatch is explained by the fact that the structural analysis in the GUSAR 2019 program is based on the 2D approach and, therefore, does not take into account steric features of the receptor that the activity of which the simulated compounds are intended to inhibit.

## 3. Discussion

In the present work, using the GUSAR 2019 program, we have modeled the quantitative structure–activity relationship for 89 TK inhibitors for HSV-1 and HSV-2 in the series of some carboxamide derivatives of 5′-amino-2′,5′-dideoxy-5-ethyluridine, N2-phenylguanine, and 2-phenylamino-6-oxopurine with general structural formulas I–VI. The modeled TK inhibitors differed quite significantly in structure and belonged to different classes of organic compounds. In particular, compounds with general structural formulas I–III had a rather high degree of similarity to thymidine in the backbone structure. Compounds with general structural formulas IV–VI were more diverse and were actually structural analogues of guanine.

The modeling resulted in the construction of 12 valid QSAR consensus models focused on predicting target properties in the form of pIC_50_. Each of these consensus models contains 20 to 100 partial regression relationships, which differ from each other by a set of descriptors. The validity of the use of structurally diverse TK inhibitors for modeling is confirmed based on the rather high numerical values of statistical criteria of the internal and external validation of QSAR models M1–M12. In particular, the high descriptive ability of the consensus models M1–M12 was confirmed based on the reliable prediction of activities performed for compound structures of four training sets using two categories of metrics: (1) metrics based on R_2_ coefficients of determination (R^2^, R^2^_0_, $\bar{R_{m}^{2}}$, CCC); and (2) metrics based on errors in predicting pIC_50_ values (root mean square error (RMSEP), mean absolute error (MAE), standard deviation (SD)).

The predictive ability of QSAR models M1–M12 was evaluated using similar statistical criteria and prediction errors. Additionally, the criteria Q^2^_F1_ and Q^2^_F2_, which are also used in the scientific literature to evaluate the predictive ability of QSAR/QSPR models, were determined. All models demonstrated rather high predictive ability in predicting target properties for both internal and external test set structures regardless of their size (95 and 100% of data).

This result is not an exception to the general rule, although it may be rather cautiously perceived by followers of the methodology of Gunch, Hammett, Taft, etc. In this context, note that the GUSAR program has been used for more than ten years since the release of its first version to build (Q)SAR/QSPR models focused on the detection and quantitative prediction of different types of biological activity. The developers of this program have repeatedly demonstrated in their publications that an important and undeniable advantage of their software product is the correct modeling of organic compounds that differ significantly in the structure and type of experimental studies. This important benefit of the GUSAR software is once again confirmed by the results of the present studies and is related to the unique algorithms used to calculate descriptors, as well as methods used to select the most significant descriptors for building the final QSAR models. In particular, the calculation of descriptors, the ideology of which is described in detail in the Section 4.1 and in the Supplementary Materials, is performed in the GUSAR program not only on the basis of whole molecules, but also on the basis of their individual structural parts, including individual atoms, as well as their various combinations. The calculation of descriptors based on the nature and properties of all atoms included in the modeled structures and their local environments is dominant in the descriptor-generation methodology, unlike the calculation of properties using whole molecular structures. This approach to the calculation of descriptors allows for common elements to be found among various organic compounds differing in the nature of cyclic and acyclic moieties, and, accordingly, expands the possibilities of QSAR modeling in general.

The ideology of the consensus approach, which actually takes into account the predictions of all partial regression relationships with a focus on their statistical weights, also significantly increases the reliability of adequate prediction of quantitative indicators of biological activity.

In addition, GUSAR program developers have repeatedly reported that QSAR models based on a diverse range of compound structures have a broader applicability in virtual screening than models based on a narrow set of multiple data. Such models allow for the identification and quantification of target properties for a broader class of organic compounds.

## 4. Computational Details

### 4.1. Computational Methodology

The QSAR analysis of HSV-1 and HSV-2 TK inhibitors was performed using the GUSAR 2019 (general unrestricted structure activity relationships) program [40,41,42,43,44,45,46,47,48,49]. In total, 12 QSAR models (M1–M12) were built.

The construction of QSAR models was performed using GUSAR 2019 software in several stages based on the training sets TrS1–TrS4. To validate these models, we used the external and internal test sets (TS1–TS2 and TS3–TS4, respectively).

### 4.2. Formation of Training and Test Sets

**Figure 9:**
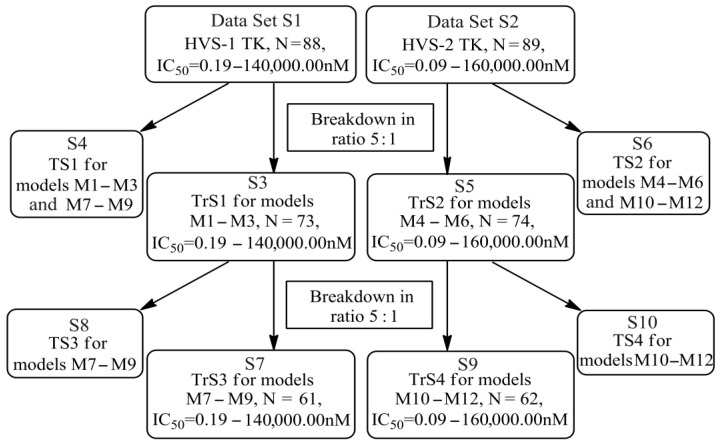
Chart of construction of the training and test sets and design of the QSAR consensus models M1–M12 (S is set, TrS and TS are training and test sets, respectively, N is the number of compounds included to the corresponding sets and arrays). Designations: (1) S1 and S2 are all datasets; (2) S3 is the training set TrS1 for models M1–M3; (3) S4 is the external test set TS1 for models M1–M3 and M7–M9; (4) S5 is the training set TrS2 for models M4–M6; (5) S6 is the external test set TS2 for models M4–M6 and M10–M12; (6) S7 is the training set TrS3 for models M7–M9; (7) S8 is the internal test set TS3 for models M7–M9; (8) S9 is the training set TrS4 for models M10–M12; (9) S10 is the internal test set TS4 for models M10–M12.

The training sets TrS1–TrS4 and external and internal test sets TS1–TS4 were formed from sets S1 and S2 according to the chart shown in Figure 9.

The datasets S1 and S2 comprised the same chemical structures (general structural formulas I–VI, Figure 1), the inhibitory activity of which against HSV-1 and HSV-2 TKs was quantitatively expressed as the IC_50_. The IC_50_ values for these compounds were determined in earlier experimental studies [17,72,73]. The minor difference between the numbers of compounds in these sets is due to elimination of one compound with an inaccurately measured IC_50_ value from the set S1 (IC_50_ > 10 μmol/L).

The training set TrS1 was designed to build QSAR models M1–M3 and included 73 HSV-1 TK inhibitors. To assess the predictive power of M1–M3, we used the external test set TS1. Both of these sets were obtained by splitting the original data set S1 in a 5:1 ratio by moving every sixth chemical compound from S1 to TS1. Previously, all structures of S1 were ranked by an increasing IC_50_ (Figure 9).

The training set TrS2 contained 74 HSV-2 TK inhibitors. It was designed to build QSAR models M4–M6. To assess the validity of these models, we used external test set TS2. Both sets were obtained from the original S2 set in the same way as TrS1 and TS1.

The training sets TrS3 and TrS4 and internal test sets TS3 and TS4 were obtained by splitting TrS1 and TrS2 in a 5:1 ratio (the chemical structures of the sets were ranked by increasing IC_50_ values). A detailed description of training sets TrS1–TrS4 and test sets TS1–TS4 is presented in Table 5 and Table 6, respectively. A comparison of the data in these tables indicated that the activity distribution of compounds in all training and test sets was almost identical. As a result, the average $\bar{pIC_{50}}$ values for HSV-1 and HSV-2 TK inhibitors were almost equal for TrS1–TrS4 and TS1–TS4.

The chemical structures of the compounds of TrS1–TrS4 and TS1–TS4 were created with the Marvin Sketch 17.22.0 program [74] and converted to the SDF format using the Discovery Studio Visualizer program [75]. To build QSAR models M1–M12, we used the IC_50_ values in mol/L, which were then converted to the pIC_50_ values:

pIC_50_ = −log_10_(IC_50_) (1)

Table 5 and Table 6 show that the scatter of IC_50_ values ΔpIC_50_ > 5 in the training sets is an important condition for constructing reliable QSAR models [76].

### 4.3. Building QSAR Models

QSAR models were developed using the GUSAR 2019 software. Chemical structures were described using three types of descriptors, the calculation of which is incorporated in this software: whole-molecule descriptors, 35 electro-topological descriptors (quantitative neighborhoods of atoms, QNAs), and 30 substructural descriptors (multilevel neighborhoods of atoms, MNAs). The whole-molecule descriptors used in GUSAR include the topological length, topological volume, and lipophilicity [40,41,42,43,44,45,46,47,48,49,55,56,57,58,59,60,61,62,63]. We will briefly explain the ideology of QNA and MNA descriptor formation, as it is rather new and unconventional in terms of classical QSAR approaches. A detailed description of the ideology of these computations is presented in the Supplementary Materials.

Formally, QNA descriptors represent the structure of a molecule using only two descriptors (P and Q). The P and Q values are calculated on the basis of the connectivity matrix (C) and atomic characteristics, such as the standard ionization potential (IP) and electron affinity (EA). The values for P and Q for each atom i are calculated as follows:

(2) $$P_{i}=B_{i}\sum_{k}{\left(\exp\left(-\frac{1}{2}C\right)\right)}_{ik}B_{k}$$

(3) $$Q_{i}=B_{i}\sum_{k}{\left(\exp\left(-\frac{1}{2}C\right)\right)}_{ik}B_{k}A_{k}$$

(4) $$A_{k}=\frac{1}{2}\left(IP_{k}+EA_{k}\right)$$

(5) $$B_{k}={\left(IP_{k}-EA_{k}\right)}^{-1/2}$$

where k stands for all the other atoms in the molecule, IP is the first ionization potential, EA is the electron affinity for each atom (in eV), and C is the connectivity matrix for the molecule as a whole [42]. The standard IP and EA values of atoms in a molecule were taken from the literature.

Bivariate Chebyshev polynomials are used for the further approximation of P and Q functions over the entire junction structure. The regression equations use the averaged values of specific bivariate Chebyshev polynomials as independent variables. The averaging of these functions takes into account all atoms that are directly bonded to at least two other neighboring atoms. A detailed account of the calculation of QNA descriptors is presented in the Supplementary Materials.

In addition, GUSAR allows for the creation of QSAR models based on different biological activity profiles that are predicted for the compounds included in the training sets. All theoretically acceptable biological activities for the compounds used to build a QSAR model can be predicted with the PASS algorithm. The current version of PASS predicts more than 6000 types of biological activity with an average prediction accuracy of about 95%. This list includes pharmacotherapeutic effects, mechanisms of action, side and toxic effects, metabolic conditions, sensitivity to transporter proteins, and gene expression related activity. Adequate performance of the PASS algorithm is realized under conditions where each structure is represented as a list of MNA descriptors. Accordingly, before running the PASS algorithm, a set of MNA descriptors is preliminarily automatically calculated for each compound [40,41,42]. Thus, it is fair to say that in this case, regression models are based on MNA descriptors. The results of the PASS procedure for each type of biological activity are outputted in the form of a list of Pa-Pi parameter values, representing the difference between the probabilities that a compound is active (Pa) or inactive (Pi), respectively. Subsequently, the selection of independent variables necessary for the construction of regression relationships is performed automatically from this list at random. A detailed account of the calculation of MNA descriptors is also presented in the Supplementary Materials.

The self-consistent regression method (SCR) was applied to select the optimal number of descriptors for the QSAR models [20,21,22]. As previously reported by the developers of the GUSAR 2019 program, this makes it possible to remove the variables that poorly describe the target value. Additionally, this method is resistant to the noise in the data [55,56,57,58,60].

The SCR method of descriptor selection is a regularized least squares method based on the use of the mathematical apparatus of Bayesian statistics for the optimal estimation of regularization parameters and descriptor selection for the subsequent construction of regression relationships. The essence of the SCR method is the iterative selection of regularization coefficients ν_i_, first, to find the optimal number of descriptors in the regression equation, and second, to find the maximum values for regression coefficients a_i_, and thus to obtain the maximum and, hence, reliable values of the dependent variable y for the training set compounds. As a result, the minimum error of quantitative prediction of the target property is achieved. Unlike the multiple linear regression method, which is traditionally used to solve such problems, the SCR method, based on its ideology, does not impose restrictions on the number of regressors in the final regression equation or on the absence of a correlation (or the presence of a weak correlation) between them. Thus, the advantages of the SCR descriptor selection method over classical multiple linear regression are obvious. Unlike various heuristic approaches that solve multiple linear regression problems, descriptor selection in the SCR method is mathematically sound. Because of this, the SCR method can be successfully applied to remove variables that poorly describe the modeled activity value, while retaining a set of variables that correctly represent the existing relationships. The detailed mathematical apparatus on which the SCR method is based is presented in previous publications [47,55,56,57,58,60] and in the Supplementary Materials.

The GUSAR 2019 program allows for the building of both partial regression dependences and consensus models based on them. In this study, to reduce the variability of the final results, we used the consensus approach to build QSAR models. The final values of statistical criteria (coefficient of determination, Fisher’s test, etc.) and the predicted pIC_50_ values for each molecule were estimated, in accordance with the consensus approach incorporated in the GUSAR 2019 software, as the weighted averages of these values derived from a set of partial QSAR models (for predictions that were within the respective areas of applicability). Meanwhile, each of these private models included in the consensus model was built independently based on a combination of QNA and/or MNA descriptors with the above three types of whole-molecule descriptors. This algorithm allowed us to combine the results of QSAR modeling based on different types of descriptors that are provided in the GUSAR 2019 software. As a result, we built 12 QSAR consensus models, which included 20 to 320 partial models. Since we used QSAR consensus models derived from dozens or even hundreds of single QSAR models, it is not possible to provide a general equation describing all selected variables. For this reason, the created QSAR consensus models could not provide information about positively and negatively influencing descriptors. Instead, GUSAR 2019 shows the positive and negative impact of each atom of the molecule on the predicted value [61]. An analysis of the effect of atoms on predicted pIC_50_ values and the search for general relationships between the structures of active compounds interacting with targets is described in this publication in Section 2.

### 4.4. Assessment of Applicability

GUSAR 2019 uses three different approaches to assess the applicability of the models: similarity, leverage, and accuracy assessment, which were described in detail previously [20,21,22,42]. A detailed description of these characteristics is given in the Supplementary Materials.

## 5. Evaluation of the Quality and Predictive Ability of QSAR Models

### 5.1. Calculating the pIC_50_ Values Using the Consensus Approach in the GUSAR 2019 Program

The descriptive and predictive ability of the M1–M12 consensus models was evaluated using the results of predicting pIC_50_ values for the structures included in the training sets TrS1–TrS4 and test sets TS1–TS4, respectively. For internal validation, a cross-validation control was used with a random twenty-fold exclusion of 20% of the structures from each training set.

### 5.2. Statistical Parameters Characterizing the Predictive Ability of QSAR Models

The predictive ability of QSAR models was estimated by predicting the pIC_50_ values for HSV-1 and HSV-2 TK inhibitors included in the external and internal test sets using two types of metrics:

(1) based on coefficients of determination R^2^ (R^2^, R^2^_0_, Q^2^_F1_, Q^2^_F2_, $\bar{R_{m}^{2}}$, CCC);
(2) estimating the prognostic errors of pIC_50_: standard error (RMSEP), mean absolute error (MAE), and standard deviation (SD) [68,69,70,71,77].

Statistical parameters were calculated using the XternalValidationPlus 1.2 program [66,78]. The relevant formulas are presented in the Supplementary Materials. The same program was used to identify systematic errors in the constructed consensus models. To avoid false predictions associated with outliers in experimental data, XternalValidationPlus 1.2 automatically removes 5% of compounds with high residuals.

In this work, when assessing the descriptive and predictive ability of the QSAR models, we mainly focused on the recommendations of Roy et al. [71]. We rated the descriptive and predictive power of each of the M_i_ QSAR models we developed as high if the following four conditions were met simultaneously:

(1) different coefficients of determination, calculated by comparing the experimental data with the calculated pIC_50_ data contained in each of the training and test sets, respectively, were numerically similar and tended to be 1;
(2) MAE values for predicted pIC_50_ of compounds of the training or test set, respectively, did not exceed 10% of the range of variation of the experimental pIC_50_ values for this set;
(3) the following relation held: MAE+3·SD_TrS_ ≤ 0.2·pIC_50 TrS_, where ΔpIC_50_ is the range of variation of pIC_50_ values for the TrS structures (this criterion refers to the assessment of the descriptive ability of the model);
(4) the following relation held: MAE+3·SD_TrS_ ≤ 0.2·pIC_50 TrS_, where ΔpIC_50_ is the range of variation of pIC_50_ values for the TrS structures (the criterion refers to the assessment of the predictive ability of the model).

We rated the descriptive and predictive ability of each of the M_i_ QSAR models we developed as low if the following conditions were met simultaneously:

(1) the numerical values of different coefficients of determination, calculated by comparing the experimental data with calculated pIC_50_, did not exceed 0.6;
(2) MAE values estimated from the results of comparing the experimental and predicted pIC_50_ values of compounds of the training or test set, respectively, did not exceed 20% of the range of variation of the experimental pIC_50_ values in the training set used to build the M_i_ model;
(3) the following relation held: MAE + 3·SD_TrS_ ≥ 0.25·pIC_50 TrS_, where ΔpIC_50_ is the range of variation of pIC_50_ values for the TrS structures (the criterion refers to the assessment of the descriptive ability of the model);
(4) the following relation held: MAE + 3·SD_TS_ ≥ 0.25·pIC_50 TrS_, where ΔpIC_50_ is the range of variation of pIC_50_ values for the TrS structures (the criterion refers to the assessment of the predictive ability of the model).

The predictions not meeting any of the above conditions are considered moderate. If the QSAR model contained a systematic error, then it was excluded from consideration.

### 5.3. Evaluation of the Contribution of Atoms to the Target Activity

QSAR consensus models M3 and M6, containing 73 and 74 HSV-1 and HSV-2 TK inhibitors, respectively, were further used for assessing the contribution of atoms and functional groups to the simulated activity. It should be noted that this procedure is automatically implemented in the GUSAR 2019 program when calculating QNA descriptors and constructing QSAR models based on them.

## 6. Conclusions

Based on the QSAR methodology using the GUSAR 2019 program, a quantitative relationship between the structure and inhibitory activity against thymidine kinase of the herpes viruses HSV-1 and HSV-2 have been found in a series of 89 derivatives of 5-ethyluridine, N2-guanine, and 6-oxopurine. The inhibitory activities of the simulated compounds were in the range of IC_50_ = 0.09–160,000.00 nmol/L. Based on the MNA and QNA descriptors and whole-molecule descriptors using the self-consistent regression, we have constructed 12 statistically significant QSAR consensus models characterized by high accuracy of the prediction of pIC_50_ values for inhibitors of thymidine kinase of the herpes viruses HSV-1 and HSV-2 (R^2^_TrS_ > 0.6; Q^2^_TrS_ > 0.5; R^2^_TS_ > 0.5). All of them can be used for virtual screening of new TK inhibitors in a series of 5-ethyluridine, N2-guanine, and 6-oxopurine derivatives.

Thus, the approach implemented in the GUSAR 2019 program makes it possible to model, with a high degree of reliability, the inhibitory activity of derivatives of 5-ethyluridine, N2-guanine, and 6-oxopurine with respect to the TK of human herpes viruses in order to develop new inhibitors of this enzyme. 

The approach implemented in the GUSAR 2019 program allows for the reliable simulation of the inhibitory activity of derivatives of 5-ethyluridine, N2-guanine, and 6-oxopurine against TK of human herpes viruses to develop new inhibitors of this enzyme. In addition, the correctness of the computational protocols used and the construction of regression models in the GUSAR 2019 program are confirmed by the absence of a systematic error in the calculations.

## Figures and Tables

**Table 1:** Statistical parameters and accuracy of the predicted pIC_50_ values of the compounds included in the training sets TrS1–TrS4 within the M1–M12 consensus models. ∆pIC_50 TrS1_ = ∆pIC_50 TrS3_ = 5.867, ∆pIC_50 TrS2_ = ∆pIC_50 TrS4_ = 6.250 ^1^.

Training Set	Model	N	N_PM_	$\bar{R^{2}}$	$\bar{F}$	$\bar{SD}$	$\bar{Q^{2}}$	V
QSAR models based on the QNA descriptors								
TrS1	M1	73	20	0.878	67.101	0.569	0.848	7
TrS2	M4	74	20	0.891	84.683	0.593	0.869	6
TrS3	M7	61	20	0.875	50.879	0.579	0.837	7
TrS4	M10	62	20	0.891	65.152	0.598	0.863	6
QSAR models based on the MNA descriptors								
TrS1	M2	73	20	0.878	63.594	0.568	0.854	7
TrS2	M5	74	20	0.906	79.140	0.552	0.887	8
TrS3	M8	61	20	0.882	51.831	0.565	0.853	7
TrS4	M11	62	20	0.894	70.947	0.589	0.872	6
QSAR models based on both QNA and MNA descriptors								
TrS1	M3	73	320	0.891	57.523	0.542	0.862	8
TrS2	M6	74	320	0.905	70.945	0.559	0.882	8
TrS3	M9	61	320	0.881	45.955	0.570	0.846	7
TrS4	M12	62	320	0.899	63.865	0.578	0.873	7

^1^ N is the number of structures in the training set; N_PM_ is the number of regression equations used for the consensus model; $\bar{R^{2}}$ is the coefficient of determination calculated for the compounds of TrSi; $\bar{Q^{2}}$ is the correlation coefficient calculated for the training set based on cross-validation with the exception of one; $\bar{F}$ is Fisher’s criterion; $\bar{SD}$ is the standard deviation; V is the number of variables in the final regression equation.

**Table 2:** Validation parameters of the QSAR models estimated using the Xternal Validation Plus 1.2 program based on the experimental and predicted pIC_50_ values of the HSV-1 TK inhibitors from training sets TrS1 (M1–M3) and TrS3 (M7–M9). ΔpIC_50 TrS1_ = ∆pIC_50 TrS3_ = 5.867 ^1^.

Comments	Prediction Parameters	QSAR Model Used for Predicting pIC_50_					
		TrS1				TrS2	
		M1	M2	M3	M7	M8	M9
Classical metrics (after removing 5% of the data with high residuals)	R^2^	0.9609	0.9594	0.9653	0.9591	0.9611	0.9654
	R^2^_0_	0.9555	0.9579	0.9614	0.9556	0.9587	0.9593
	R^2′^_0_	0.8443	0.8804	0.8661	0.8568	0.8725	0.8483
	$\bar{R_{m}^{2}}$	0.8776	0.9052	0.8952	0.8883	0.8971	0.8819
	∆$\bar{R_{m}^{2}}$	0.0379	0.0355	0.0326	0.0379	0.0352	0.0342
	CCC	0.9755	0.9777	0.9790	0.9759	0.9779	0.9775
Mean absolute error and standard deviation for the test set (after removing 5% of the data with high residuals)	RMSE	0.3368	0.3331	0.3193	0.3323	0.3327	0.3331
	MAE	0.2914	0.2784	0.2673	0.2872	0.2768	0.2830
	SD	0.1701	0.1844	0.1758	0.1687	0.1861	0.1773
	MAE + 3·SD	0.8016	0.8314	0.7948	0.7933	0.8351	0.8149
Prediction quality	-	Good					
Presence of systematic errors	-	Absent					

^1^ R^2^, R^2^_0_, and R^2′^_0_ are the determination coefficients calculated with and without taking into account the origin; average $\bar{R_{m}^{2}}$ is the averaged determination coefficient of the regression function calculated using the determination coefficients on the ordinate axis (R^2^_m_) and on the abscissa axis (R^2′^_m_), respectively; ∆$\bar{R_{m}^{2}}$ is the difference between R^2^_m_ and R^2′^_m_; CCC is the concordance correlation coefficient; MAE is the mean absolute error; SD is the standard deviation.

**Table 3:** Validation parameters of the QSAR models estimated using the Xternal Validation Plus 1.2 program based on the experimental and predicted pIC_50_ values of the HSV-2 TK inhibitors from training sets TrS2 (M4–M6) and TrS4 (M10–M12). ΔpIC_50 TrS2_ = ∆pIC_50 TrS4_ = 6.250 ^1^.

Comments	Prediction Parameters	QSAR Model Used for Predicting pIC_50_					
		TrS2			TrS4		
		M4	M5	M6	M10	M11	M12
Classical metrics (after removing 5% of the data with high residuals)	R^2^	0.9714	0.9712	0.9719	0.9708	0.9676	0.9743
	R^2^_0_	0.9687	0.9701	0.9694	0.9681	0.9664	0.9710
	R^2′^_0_	0.8890	0.9086	0.8927	0.8889	0.9009	0.8891
	$\bar{R_{m}^{2}}$	0.9137	0.9267	0.9142	0.9148	0.9216	0.9109
	∆$\bar{R_{m}^{2}}$	0.0270	0.0260	0.0267	0.0273	0.0290	0.0252
	CCC	0.9830	0.9843	0.9836	0.9827	0.9823	0.9844
Mean absolute error and standard deviation for the test set (after removing 5% of the data with high residuals)	RMSE	0.3278	0.3121	0.3146	0.3333	0.3328	0.3164
	MAE	0.2712	0.2590	0.2624	0.2739	0.2822	0.2676
	SD	0.1856	0.1753	0.1748	0.1915	0.1781	0.1703
	MAE + 3·SD	0.8279	0.7850	0.7868	0.8484	0.8164	0.7785
Prediction quality	-	Good					
Presence of systematic errors	-	Absent					

^1^ R^2^, R^2^_0_, and R^2′^_0_ are the determination coefficients calculated with and without taking into account the origin; average $\bar{R_{m}^{2}}$ is the averaged determination coefficient of the regression function calculated using the determination coefficients on the ordinate axis (R^2^_m_) and on the abscissa axis (R^2′^_m_), respectively; ∆$\bar{R_{m}^{2}}$ is the difference between R^2^_m_ and R^2′^_m_; CCC is the concordance correlation coefficient; MAE is the mean absolute error; SD is the standard deviation.

**Table 4:** Potential effective HSV-1 and HSV-2 TK inhibitors selected from the ChEMBL database using virtual screening with QSAR models M3 and M6.

No.	Name in ChEBIL	Structure			pIC_50pred_		Selectivity $\left(Selectivity=\frac{IC_{50,HSV-1}}{IC_{50,HSV-2}}\right)$
					HSV-1	HSV-2	
		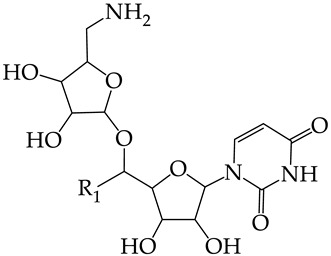					
		R_1_					
**1**	CHEMBL1199108	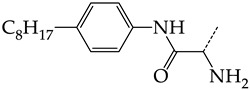			15.29	2.87	5.3359
**2**	CHEMBL1199070	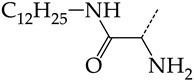			32.52	13.98	2.3267
**3**	CHEMBL1199059	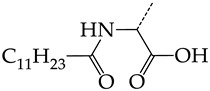			27.75	21.38	1.2980
**4**	CHEMBL1780207	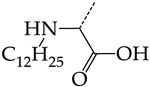			30.42	21.46	1.4176
		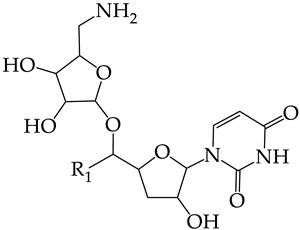					
		R_1_					
**5**	CHEMBL20028	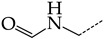			35.85	27.30	1.3131
**6**	CHEMBL1178256	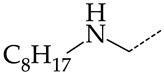			31.91	5.91	5.4029
**7**	CHEMBL19326	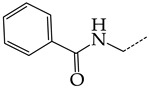			14.87	3.82	3.8897
**8**	CHEMBL1178302	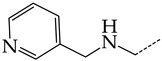			13.77	3.27	4.2105
**9**	CHEMBL19510	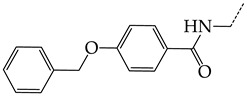			9.73	1.37	7.0878
**10**	CHEMBL1178307 *	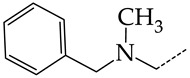			13.97	2.63	5.3210
**11**	CHEMBL19608	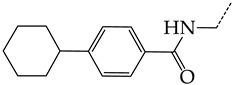			6.88	0.83	8.3308
**12**	CHEMBL19725	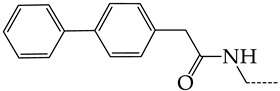			10.33	2.06	5.0177
**13**	CHEMBL19782	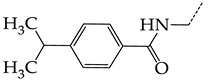			8.01	1.41	5.6706
**14**	CHEMBL1178314	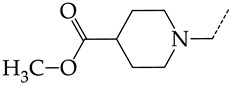			8.42	1.52	5.5286
**15**	CHEMBL1178315	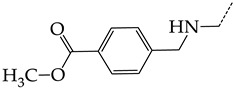			9.27	1.73	5.3491
**16**	CHEMBL277025	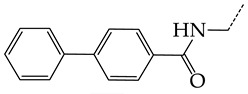			12.04	1.51	7.9804
**17**	CHEMBL1183046	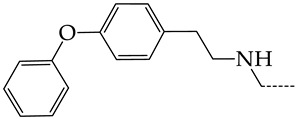			11.01	0.99	11.0940
**18**	CHEMBL277844	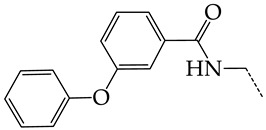			5.76	0.70	8.2058
**19**	CHEMBL1183063	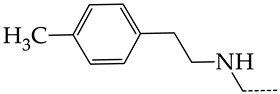			12.58	2.05	6.1317
**20**	CHEMBL278626	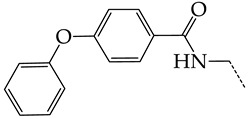			8.87	0.89	9.9477
**21**	CHEMBL1183081	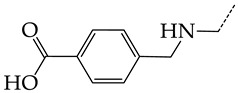			12.39	2.53	4.9020
**22**	CHEMBL1183082	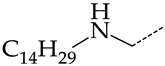			34.36	7.19	4.7770
**23**	CHEMBL1183089	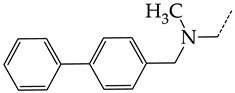			10.95	1.20	9.1477
**24**	CHEMBL1183095	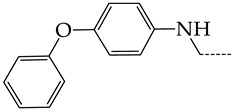			8.78	0.71	12.4135
**25**	CHEMBL1183096	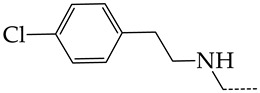			7.31	1.04	7.0456
**26**	CHEMBL279892	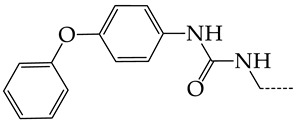			8.78	0.74	11.7868
**27**	CHEMBL1183107	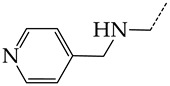			14.50	4.72	3.0716
**28**	CHEMBL1183108	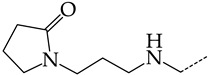			13.71	3.16	4.3415
**29**	CHEMBL280909	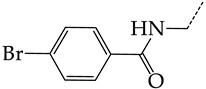			5.38	1.07	5.0082
**30**	CHEMBL1183123	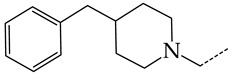			8.20	0.83	9.8336
**31**	CHEMBL1183154	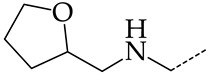			15.30	4.26	3.5958
**32**	CHEMBL1183178	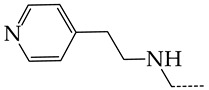			11.23	2.77	4.0530
**33**	CHEMBL1183185	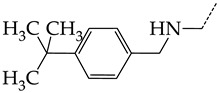			8.32	1.57	5.2872
**34**	CHEMBL1185346	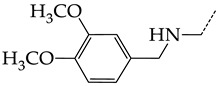			8.28	1.06	7.7791
**35**	CHEMBL1185463	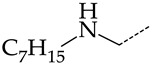			32.63	6.28	5.1918
**36**	CHEMBL1185716	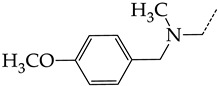			8.81	0.93	9.4314
		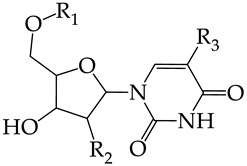					
		R_1_	R_2_	R_3_			
**37**	CHEMBL217675 *	-H	-H	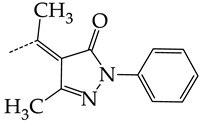	62.83	26.96	2.3306
**38**	CHEMBL238635	-H	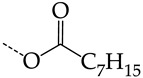	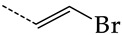	36.62	42.98	0.8520
**39**	CHEMBL2403290 *	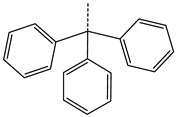	-H	-CH_3_	26.44	40.28	0.6564
**40**	CHEMBL241407 *	-H	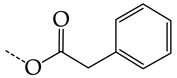	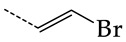	14.48	22.16	0.6535
**41**	CHEMBL241408 *	-H	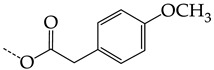	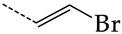	10.05	6.47	1.5544
**42**	CHEMBL1183075 *	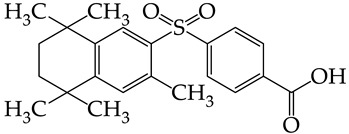			15.18	3.11	4.8817

The asterisk marks structures that obey Lipinski’s rule of five.

**Table 5:** Statistical characteristics of the training sets TrS1–TrS4.

Designation of TrS_i_	Code of the Training Set			
	HSV-1		HSV-2	
	TrS1	TrS3	TrS2	TrS4
N	73	61	74	62
$\bar{pIC_{50}}$	6.788		6.921	
∆pIC_50_	5.867		6.250	
Thresholds used to evaluate the model’s forecast				
0.10 × ∆pIC_50_	0.587		0.625	
0.15 × ∆pIC_50_	0.880		0.938	
0.20 × ∆pIC_50_	1.174		1.250	
0.25 × ∆pIC_50_	1.467		1.563	

**Table 6:** Statistical characteristics of the test sets TS1–TS4.

Designation of TS_i_	Code of the Test Set			
	HSV-1		HSV-2	
	TS1	TS3	TS2	TS4
N	15	12	15	12
$\bar{pIC_{50}}$	6.788		6.921	
∆pIC_50_	5.867		6.250	
Distribution of the observed response values of test sets TSi around the test mean				
$\bar{pIC_{50}}$ ± 0.5, %	26.667	16.667	20.000	25.000
$\bar{pIC_{50}}$ ± 1.0, %	40.000	41.667	40.000	41.667
$\bar{pIC_{50}}$ ± 1.5, %	60.000	58.333	46.667	50.000
$\bar{pIC_{50}}$ ± 2.0, %	73.333	83.333	66.667	66.667
Distribution of the observed response values of test sets TSi around the training mean				
$\bar{pIC_{50}}$ ± 0.5, %	13.333	8.333	26.667	16.667
$\bar{pIC_{50}}$ ± 1.0, %	33.333	25.000	33.333	41.667
$\bar{pIC_{50}}$ ± 1.5, %	46.667	50.000	46.667	50.000
$\bar{pIC_{50}}$ ± 2.0, %	66.667	75.000	66.667	75.000

## Data Availability

Data are contained within the article and supplementary materials.

## Supplementary Materials

The following supporting information can be downloaded at: https://www.mdpi.com/article/10.3390/molecules28237715/s1, Supplementary file contains Tables S1–S14. Table S1: The equations for assessing the descriptive and predictive potentials of the QSAR models based on the R^2^ and MAE metrics; Table S2: The validation parameters of the QSAR models estimated using the Xternal Validation Plus 1.2 program based on the experimental and predicted values of the HSV-1 TK inhibitors from test set TS1; Table S3: The validation parameters of the QSAR models estimated using the Xternal Validation Plus 1.2 program based on the experimental and predicted data for HSV-2 TK inhibitors from internal test set TS2; Table S4: The validation parameters of the QSAR models estimated using the Xternal Validation Plus 1.2 program based on the experimental and predicted data for the HSV-1 TK inhibitors from test set TS3; Table S5: The validation parameters of the QSAR models estimated using the Xternal Validation Plus 1.2 program based on the experimental and predicted data for the HSV-2 TK inhibitors from internal test set TS4; Table S6: Prediction of the pIC_50_ values for the TrS1 compounds using models M1–M3; Table S7: Prediction of the pIC_50_ values for the TrS2 compounds using models M4–M6; Table S8: Prediction of the pIC_50_ values for the TrS3 compounds using models M7–M9; Table S9: Prediction of the pIC_50_ values for the TrS4 compounds using models M10–M12; Table S10: Prediction of the pIC_50_ values for the TS1 compounds using models M1–M3, M7–M9; Table S11: Prediction of the pIC_50_ values for the TS2 compounds using models M4–M6, M10–M12; Table S12: Prediction of the pIC_50_ values for the TS3 compounds using models M7–M9; Table S13: Prediction of the pIC_50_ values for the TS4 compounds using models M10–M12; Table S14: Potential effective inhibitors of thymidine kinase of human herpes viruses HSV-1 and HSV-2 selected from the ChEMBL database using virtual screening with QSAR models M3 and M6.
