# Supplementary material for: Quantitative Structure–Activity Relationship in the Series of 5-Ethyluridine, N2-Guanine, and 6-Oxopurine Derivatives with Pronounced Anti-Herpetic Activity
Source: Molecules. 2023 Nov 22;28(23):7715. doi: 10.3390/molecules28237715 (PMC10708366; doi:10.3390/molecules28237715)
Supplement: Supplementary file 1 [file molecules-28-07715-s001.zip › molecules-2665158-supplementary.pdf]

Quantitative Structure–Activity Relationship in the Series of 5-Ethyluridine, N2-Guanine, and 6-Oxopurine Derivatives with Pronounced Anti-Herpetic Activity

Veronika Khairullina * and Yuliya Martynova

Institute of Chemistry and Defence in Emergency Situations, Ufa University of Science and Technology, 50076 Ufa, Russia; martynovayuz@uust.ru
* Correspondence: khajrullinavr@uust.ru; Tel.: +7-963-906-6567

Supplementary Material

CONTENTS

PARAMETERS FOR ASSESSING THE DESCRIPTIVE AND PREDICTIVE POTENTIAL OF QSAR MODELS 3

2. BRIEF DESCRIPTION OF THE GUSAR 2019 PROGRAM 6

2.1. CALCULATION OF STRUCTURAL DESCRIPTORS 6

2.2. SELECTION OF THE DESCRIPTORS WHEN CONSRTUCTING QSAR MODELS. 10

2.3. CONSTRUCTING QSAR MODELS 13

2.4. ASSESSMENT OF THE RANGE OF APPLICABILITY 14

3. RESULTS 15

PARAMETERS FOR ASSESSING THE DESCRIPTIVE AND PREDICTIVE POTENTIAL OF QSAR MODELS

Table S1. The equations for assessing the descriptive and predictive potentials of the QSAR models based on the R^2 and MAE metrics

Comment	Equation of criterion	
Parameters for assessing the descriptive and predictive potential of QSAR models using internal cross-validation techniques		
Determination coefficient (Coefficient of multiple determination R^2) is the determination coefficient of the calculated using the experimental and the predicted data of the training set	$R^2 = 1 - \frac{\sum_{i=1}^{N_{TrSi}}(y_i^{pred} - y_i^{obs})^2}{\sum_{i=1}^{N_{TrSi}}(y_i^{obs} - \overline{y^{obs}})^2} = 1 - \frac{RSS}{TSS};$ $R^2 = \left(\frac{\sum_{i=1}^{N_{TrSi}}(y_i^{obs} - \overline{y^{obs}})(y_i^{pred} - \overline{y^{pred}})}{\sqrt{\sum_{i=1}^{N_{TrSi}}(y_i^{obs} - \overline{y^{obs}})^2 \times \sum_{i=1}^{N_{TrSi}}(y_i^{pred} - \overline{y^{pred}})^2}}\right)^2$	(1)
R_0^2 and $R_0'^2$ are respectively the determination coefficients of the calculated using the experimental and the predicted data of the training set, forcing respectively the origin of the axis	$R_0^2 = 1 - \frac{\sum_{i=1}^{N_{TrSi}}\left(y_i^{pred} - k * y_i^{pred}\right)^2}{\sum_{i=1}^{N_{TrSi}}\left(y_i^{pred} - \overline{y^{pred}}\right)^2};$ $R_0'^2 = 1 - \frac{\sum_{i=1}^{N_{TrSi}}\left(y_i^{obs} - k' * y_i^{obs}\right)^2}{\sum_{i=1}^{N_{TrSi}}\left(y_i^{obs} - \overline{y^{obs}}\right)^2};$ $k = \frac{\sum_{i=1}^{N_{TrSi}}(y_i^{obs} * y_i^{pred})}{\sum_{i=1}^{N_{TrSi}}(y_i^{pred})^2}; k' = \frac{\sum_{i=1}^{N_{TrSi}}(y_i^{obs} * y_i^{pred})}{\sum_{i=1}^{N_{TrSi}}(y_i^{obs})^2}$	(2)
R^2_m is determination coefficient of the regression function, calculated using the experimental values on the ordinate axis, R'^2_m using them on the abscissa	$R_m^2 = R_{TrSi}^2\left(1 - \sqrt{R_{TrSi}^2 - R_0^2/_{TrSi}}\right) > 0.5;$ $\Delta R_m^2 = [R_m^2 - R'^2_m] < 0.2;$ $\overline{R_m^2} = \frac{R_m^2 + R'^2_m}{2}$	(3)
Determination coefficient by internal cross-validation	$Q^2 = Q^2_{20\%(n=20)} = 1 - \frac{\sum_{i=1}^{N_{TrSi}}\left(y_{i/i}^{pred} - y_i^{obs}\right)^2}{\sum_{i=1}^{N_{TrSi}}\left(y_i^{obs} - \overline{y^{obs}}\right)^2}$ $= 1 - \frac{PRESS}{TSS}$	(4)

Standard deviation	$$S.D. = \sqrt{\frac{\sum_{i=1}^{N_{TrSi}}\left(y_i^{obs} - y_i^{pred}\right)^2}{N_{TrSi} - V - 1}} = \sqrt{\frac{RSS}{N_{TrSi} - V - 1}}$$	(5)
Root Mean Square Error in in prediction activity for training set	$$RMSE = \sqrt{\frac{\sum_{i=1}^{N_{TrSi}}\left(y_i^{obs} - y_i^{pred}\right)^2}{N_{TrSi}}} = \sqrt{\frac{RSS}{N_{TrSi}}}$$	(6)
Variance ratio (F)	$$F = \frac{\sum_{i=1}^{N_{TrSi}}\left(y_i^{pred} - \overline{y^{obs}}\right)^2}{\sum_{i=1}^{N_{TrSi}}\left(y_i^{obs} - y_i^{pred}\right)^2} \times \frac{N_{TrSi} - V - 1}{V}$$	(7)
Parameters for estimating the predictive power of QSAR models using external cross-validation technique		
R_0^2 and $R_0'^2$ are calculated forcing the regression line to pass through the origin, k and k' are the slope of the regression lines	$$R_0^2 = 1 - \frac{\sum_{i=1}^{N_{TSi}}\left(y_i^{pred} - k * y_i^{pred}\right)^2}{\sum_{i=1}^{N_{TSi}}\left(y_i^{pred} - \overline{y^{pred}}\right)^2};$$ $$R_0'^2 = 1 - \frac{\sum_{i=1}^{N_{TSi}}\left(y_i^{obs} - k' * y_i^{obs}\right)^2}{\sum_{i=1}^{N_{TSi}}\left(y_i^{obs} - \overline{y^{obs}}\right)^2};$$ $$k = \frac{\sum_{i=1}^{N_{TSi}}\left(y_i^{obs} * y_i^{pred}\right)}{\sum_{i=1}^{N_{TSi}}\left(y_i^{pred}\right)^2};$$ $$k' = \frac{\sum_{i=1}^{N_{TSi}}\left(y_i^{obs} * y_i^{pred}\right)}{\sum_{i=1}^{N_{TSi}}\left(y_i^{obs}\right)^2}$$	(8)
Correlation coefficient between observed and predicted activities	$$R_{TSi}^2 = 1 - \frac{\sum_{i=1}^{N_{TSi}}(y_i^{pred} - y_i^{obs})^2}{\sum_{i=1}^{N_{TSi}}(y_i^{obs} - \overline{y^{obs}})^2} = 1 - \frac{PRESS}{TSS};$$ $$R_{TSi}^2 = \left(\frac{\sum_{i=1}^{N_{TSi}}(y_i^{obs} - \overline{y^{obs}})(y_i^{pred} - \overline{y^{pred}})}{\sqrt{\sum_{i=1}^{N_{TSi}}\left(y_i^{obs} - \overline{y^{obs}}\right)^2 \times \sum_{i=1}^{N_{TSi}}\left(y_i^{pred} - \overline{y^{pred}}\right)^2}}\right)^2$$	(9)

The determination coefficients calculated for the connections of the test sample TS_i, taking into account the mean pIC_{50} for the training samples, the mean pIC_{50} for the test samples, respectively	$Q^2_{F_1} = 1 - \frac{\sum_{i=1}^{N_{TSi}}\left(y_i^{pred} - y_i^{obs}\right)^2}{\sum_{i=1}^{N_{TSi}}\left(y_i^{obs} - \overline{y_{i/TrSi}^{obs}}\right)^2} = 1 - \frac{PRESS}{TSS_{test}(\overline{y_{i/TrSi}^{obs}})}$	(10)
	$Q^2_{F_2} = 1 - \frac{\sum_{i=1}^{N_{TSi}}\left(y_i^{pred} - y_i^{obs}\right)^2}{\sum_{i=1}^{N_{TSi}}\left(y_i^{obs} - \overline{y_{i/TSi}^{obs}}\right)^2} = 1 - \frac{PRESS}{TSS_{test}\left(\overline{y_{i/TSi}^{obs}}\right)} = R^2_{TSi}$	(11)
Concordance Correlation Coefficient (CCC)	$CCC = \frac{2\sum_{i=1}^{N_{TSi}}(y_i^{obs} - \overline{y^{obs}})(y_i^{pred} - \overline{y^{pred}})}{\sum_{i=1}^{N_{TSi}}(y_i^{obs} - \overline{y^{obs}})^2 + \sum_{i=1}^{N_{TSi}}(y_i^{pred} - \overline{y^{pred}})^2 + N_{TSi}(\overline{y^{obs}} - \overline{y^{pred}})}$	(12)
R^2_m is determination coefficient of the regression function, calculated using the experimental values on the ordinate axis, R'^2_m using them on the abscissa	$R^2_m = R^2_{TSi}\left(1 - \sqrt{R^2_{TSi} - R^2_{0TSi}}\right) > 0.5;$ $\Delta R^2_m = [R^2_m - R'^2_m] < 0.2;$ $\overline{R^2_m} = \frac{R^2_m + R'^2_m}{2}$	(13)
Root Mean Square Error in prediction activity for test set	$RMSEP = \sqrt{\frac{\sum_{i=1}^{N_{TSi}}\left(y_i^{obs} - y_i^{pred}\right)^2}{N_{TSi}}} = \sqrt{\frac{RSS}{N_{TSi}}}$	(14)
Mean Absolute Error	$MAE = \frac{\sum_{i=1}^{N_{TSi}}\left\lvert y_i^{obs} - y_i^{pred}\right\rvert}{N_{TSi}}$	(15)

where
TrSi is the training set, TSi is the test set,
N_{train} and N_{test} are total number of objects in the training set and test set respectively;
y_i^{obs} are experimental data values, y_i^{pred} are predicted data values,
$\overline{y^{obs}}$ are average of the experimental data values;
$\overline{y^{pred}}$ are average of the predicted data values;
RSS is residual sum of squares;
PRESS is the sum of the squares of the prediction errors (predictive sum of squares);
TSS is the total sum of squares (is sum of squared deviations from the data set mean); $TSS_{test}(\overline{y_{i/train}^{obs}})$ and $TSS_{test}(\overline{y_{i/test}^{obs}})$ are the total sum of squares of the external set calculated using the training set mean and external set mean, respectively.

2. Brief Description of the Gusar 2019 Program

2.1. calculation of structural descriptors

Here is a description of the GUSAR program necessary to understand the text of the article.

A detailed description of the ideology of calculating descriptors and constructing QSAR models using this program is given in the articles listed in the list of references and in the site http://www.pharmaexpert.ru (http://www.pharmaexpert.ru/passonline/downloads/articles/Filimonov-and-Poroikov-Chapter-6.pdf).

From a general point of view, the assessment of the activity of an organic molecule in the GUSAR2013 program is carried out according to the equation (1):

$$y_{pred} = a_0 + \sum_i a_i f_i(S) \, , \quad (1)$$

where a_0, a_1, … different functions of organic molecule's structure S.

In classic QSAR methods, the functions $f_1(S)$, $f_2(S)$, … represent physical-chemical parameters or other quantitative characteristics of molecular structure, and the coefficients a0, a1, ... are determined using multiple linear regression (MLR), partial least squares (PLS) analysis, or support vector regression (SVR), etc. QSAR methods based on the similarity between a certain molecule S_i with known biological activity and the molecule S use the value fiðSÞ of their similarity.

In the GUSAR 2019 program, the description of the structure and the calculation of the regression coefficients for the further construction of QSAR models is based on the use of two types of substructural descriptors of atomic neighborhoods: MNA (Multilevel Neighborhoods of Atoms) and QNA (Quantitative Neighborhoods of Atoms) [40,43]. They are automatically deduced from the matrices of molecular connectivity, standard ionization potentials (IP) and electron affinities (EA). The QNA descriptors are defined by two functions, P and Q. The P and Q values for each atom *i* are calculated using the following formulae [40]:

$$\mathrm{P_i} = \mathrm{B_i} \sum_{\mathrm{k}} \left(\exp\left(-\frac{1}{2}\mathrm{C} \right) \right)_{\mathrm{ik}} \mathrm{B_k} \quad (2)$$

$$\mathrm{Q_i} = \mathrm{B_i} \sum_{\mathrm{k}} \left(\exp\left(-\frac{1}{2}\mathrm{C} \right) \right)_{\mathrm{ik}} \mathrm{B_k A_k} \quad (3)$$

$$\mathrm{A_k} = \frac{1}{2}(\mathrm{IP_k} + \mathrm{EA_k}), \;\; \mathrm{B_k} = (\mathrm{IP_k} - \mathrm{EA_k})^{-1/2} \quad (4)$$

where *k* is the remaining atoms in the molecule, IP is the first ionization potential, EA is the electron affinity for each atom (in eV), and C is the connectivity matrix for the molecule as a

whole [67]. The standard values IP and EA of atoms in a molecule were collected from the literature. Although the value μP-Q can be considered by convention as the partial atomic charge, where μ is the chemical potential, in general the P and Q values are not the estimate of partial atomic charges or hardness, etc.

Any atom influences the others, although the influence decreases with the increase of the distance between them. The algorithm of the QNA descriptor calculation is really very simple due to the uselessness of the matrix Exp(-1/2C) itself, the fact that the product of Exp(-1/2C) by a vectoris needed only, and the fact that the matrix C consists of 0 and 1 only. A detailed description of QNA descriptors is represented in [42].

Thus, the QNA descriptors are calculated taking into account the relationships between all atoms of the structure. These values describe each atom of the molecule but, at the same time, depend on the structure of the molecule as a whole [41].

In the future, based on the functions P and Q, the $f_i(S)$ functions are calculated. Each function of the structure of the molecule fi (S) is calculated according to equation (4) as the average value of the function gi (P, Q) for those m atoms of the molecule that have two or more immediate neighbors:

$$f_i(S) = \frac{1}{m}\sum_k g_i(P_k, Q_k) \quad (5)$$

Substitution of expression (5) into equation (1) and permutation of the sums allows one to obtain equation (6):

$$y_{pred} = a_0 + \sum_i a_i \frac{1}{m}\sum_k g_i(P_k, Q_k) = \frac{1}{m}\sum_k \left(a_0 + \sum_i a_i g_i(P_k, Q_k)\right) \quad (6)$$

Thus, in accordance with equation (6), the estimate of the parameter y_{pred} for a molecule is the average of the predicted values for specific atoms in the molecule. Formally, QNA descriptors represent the structure of a molecule with only two descriptors (P and Q), in contrast to the many traditional descriptors used in QSAR.

However, the developers of the GUSAR program found that the P and Q values are highly correlated with each other (r = 0.903). Since the values of P and Q have different scales (standard deviations are 0.023 and 0.208, respectively), the developers of the GUSAR program carried out normalization to optimize the family of functions g_i (P, Q). Normalization was performed by calculating mean values (E_P and E_Q), standard deviations (D_P and D_Q), and correlation between P and Q values (R_{PQ}):

$$P' = \frac{P - E_p}{D_p} \quad Q' = \frac{Q - E_Q}{D_Q} \quad (7)$$

$$u = \frac{P' + Q'}{\sqrt{2(1 + R_{PQ})}} \qquad u = \frac{P' - Q'}{\sqrt{2(1 - R_{PQ})}} \tag{8}$$

The orthonormal U and V have zero mean, unit variance, and they are uncorrelated [45,46].

The QNA values are the basic information for calculating the Chebyshev 2D polynomials.

$$g_i(P,Q) = T_{uv}(P,Q) = \cos(u \bullet \arccos(TanH(u))) \bullet \cos(v \bullet \arccos(TANH(v))) \tag{9}$$

where the integers u, v=0, 1, 2, ... define the 2D Chebyshev polynomial degree. The final equation for estimate y_{pred} using QNA descriptors is

$$y_{pred} = \frac{1}{m}\sum_k \left(a_0 + \sum_{uv} a_{uv} T_{uv}(P_k, Q_k)\right) = a_0 + \sum_{uv} a_{uv} T_{uv}$$
$$T_{uv} = \frac{1}{m}\sum_k \left(T_{uv}(P_k, Q_k)\right) \tag{10}$$

Thus, the regression equations constructed in the GUSAR 2019 program take into account both the specificity and physicochemical properties of each atom entering the training set [40–43]. However, QNA descriptors cannot be physically interpreted due to the peculiarities of their calculation. In this regard, they are not explicitly displayed under calculations.

The MNA descriptors are computed using the PASS algorithm (Prediction of Activity Spectra for Substances) [40,43], which predicts approximately 6,400 "biological activities" with an accuracy threshold of an average prediction of at least 95%. These descriptors are generated based on the structural formulae of chemical compounds without using any pre-compiled list of structural fragments [40–43]. The authors of the GUSAR 2019 program report that "MNA-descriptors are based on the molecular structure representation, which includes hydrogens according to the valences and partial charges of other atoms and does not specify the types of bonds." They are generated as "a recursively defined sequence:

• zero-level MNA descriptor for each atom is the mark A of the atom itself;

• any next-level MNA descriptor for the atom is the substructure notation A (D1D2…Di…), where Di is the previous-level MNA descriptor for i–th immediate neighbor of the atom A.

The neighbor descriptors $D_1D_2 \ldots D_i \ldots$ are arranged in a unique manner. This may be, for example, a lexicographic sequence. MNA descriptors are generated using an iterative procedure, which results in the formation of structural descriptors that include the first, second, etc. neighborhoods of each atom. The label contains not only information about the type of

atom, but also additional information about its belonging to a cyclic or acyclic system, etc. For example, an atom that does not enter a ring is marked with a "—".

Based on the MNA descriptors using B-statistics, calculated in the PASS program, the biological activity spectrum of a chemical compound is predicted [40–43].

The output of the PASS program is the probabilities of the activity (Pa) of inactivity (Pi) of each prognostic result. The difference between these two values (Pa–Pi) for a randomly selected subset of predicted activities is used as independent variables for regression analysis in GUSAR. GUSAR 2019 incorporates a PASS version that pedicts 4130 types of biological activity. The developers of the GUSAR 2019 program report that the list of predictable biological activities currently includes 501 pharmacotherapeutic effects, 3295 mechanisms of action, 57 adverse and toxic effects, 199 metabolic terms, 49 transporter proteins and 29 activities related to gene expression [46]. The average accuracy of a reliable prediction of biological activity, calculated by leave-one-out cross-validation procedure is approximately 95% [66].

However, the regression equation constructed based on the MNA descriptors reveals the specificity of the action of the compound but does not explicitly reflects the physicochemical parameters of chemical compounds [64,78].

In addition, the GUSAR 2019 program calculates the QSAR descriptors of an entire molecule such as topological length, topological volume, lipophilicity, and physicochemical descriptors (numbers of positive and negative charges, number of donors and acceptors of the hydrogen bond, number of aromatic atoms, molecular weight and number of halogen atoms) [40–43]. Therefore, these parameters were added to the QNA descriptors. The topological length of a molecule was calculated as the maximal distance between any two atoms and the volume of a molecule as the sum of each atom's volume, $4/3\pi R^3$, where R is the atomic radius.

The authors of the GUSAR 2019 program report that "in GUSAR, the scale of QNA- and PASS-based descriptors ranges from −1 to 1. Therefore, no additional normalization is required for these types of descriptors. Only whole-molecule descriptors are normalized using a standard Z-score normalization procedure" [40].

It should be noted that the program is able to construct QSAR models both relying solely on one of these types of descriptors, and on their combination in terms of the consensus approach [20-22]. At the same time, based on the consensus approach methodology, models for quantitative prediction of biological activity for these descriptors are calculated independently of each other. The examples of the sample QSAR GUSAR models for predicting the toxic

effects of chemical compounds are available free *via* the link http://www.way2drug.com/GUSAR.

However, it noteworthy that the features of the QNA and MNA calculations retain these descriptors without unambiguous physical interpretation. For this reason, in the commercial and academic versions of the GUSAR 2019 program for broad use, the regression equations are not displayed.

2.2. SELECTION OF THE DESCRIPTORS WHEN CONSRTUCTING QSAR MODELS

In GUSAR 2019, three approaches are used when selecting the optimal number of descriptors for constructing QSAR-models:

1) self-consistent regression method (SCF) [20-22, 40–43];

2) method of radial basis functions (RBF) [40];

3) method based on the combination of SCF and RBF [40].

The SCF and SCF-RBF methods are the most preferable. The SCF method is correctly applied to modeling compounds with a rather high degree of similarity. The other two methods of selecting the optimal number of descriptors show good results when modeling structurally dissimilar compounds.

It is obvious that any regressor has only a restricted influence on the response, i.e. large values of regression coefficients are prohibitive, i.e. they have small probabilities. We therefore suggest using an a priori probability distribution of the regression coefficients p(a|v), where v are the distribution parameters. Therefore, the estimate of a is obtained by the maximum a posteriori probability method

$$\boldsymbol{a} = \arg\max \mathrm{p}(\boldsymbol{a}|\boldsymbol{X}, \boldsymbol{y}, \boldsymbol{v}) \tag{11}$$

where p(a|***X*****,** ***y*****,** ***v***) is calculated by Bayes formula:

$$p(\boldsymbol{a}|\boldsymbol{X}, \boldsymbol{y}, \boldsymbol{v}) = \frac{p(\boldsymbol{y}|\boldsymbol{X}, \boldsymbol{a})p(\mathrm{a}|\boldsymbol{v})}{p(\boldsymbol{y}|\boldsymbol{X}, \boldsymbol{v})} \tag{12}$$

and the likelihood function of the sample p(**y**|**X,v**) is calculated by summation(integration) of all possible values of the regression coefficients **a:**

$$p(\boldsymbol{y}|\boldsymbol{X}, \boldsymbol{v}) = \sum_{\mathrm{a}} p(\boldsymbol{y}|\boldsymbol{X}, \boldsymbol{a})p(\mathrm{a}|\boldsymbol{v}) \tag{13}$$

If the residuals $\boldsymbol{\varepsilon} = \boldsymbol{y} - \boldsymbol{Xa}$ are normally distributed and an a priori conditional probability ***p(a|v)*** has also normal density:

$$p(\boldsymbol{a}|\boldsymbol{v}) \sim \exp\left[-\frac{(v_1 a_1^2 + \ldots + v_m a_m^2)}{2}\right] \tag{14}$$

It was previously shown [20–22,40–43] that self-consistent regression (SCR) can be successfully applied to various QSAR problems. The SCR method is resistant to noise in the data and allows deleting the variables that poorly describe the target value. This is a regularized method of the least squares. Independent parameters *a* are calculated in this method according to the equation (15) [40]:

$$a = \mathrm{ArgMin}\left[(\sum_{i=1}^{n} y_i - \sum_{k=0}^{m} x_{ik} a_k)^2 + \sum_{k=1}^{m} v_k a_k^2 \right] \quad (15)$$

where *a* is the regression coefficient, *n* is the number of objects, y_i is the response value of the *i*-th object, *m* is the number of independent variables, x_{ik} is the value of the *k*-th independent variable of the *i*-th object, a_k is the *k*-th value of the regression coefficients, and v_k is the *k*-th value of the regularization parameters. Equation (15) has the following solution:

$$a = TX^T y,\ \ \mathrm{Var}(a) = \sigma^2 T, T = (X^T X + \sigma^2 V)^{-1} \quad (16)$$

where X^T is the transposed regression matrix X, and $\sigma^2 V$ is the diagonal matrix of the regularization parameters. The regression coefficients obtained from the SCR reflect the contribution of each particular descriptor (variable) to the final equation. The higher the absolute value of the coefficient, the greater its contribution. Thus, the regression coefficients obtained after the SCR can be used to weight the descriptors (variables) depending on their importance.

Since the parameters v use the same data sample, X and y, we called the method ‘‘self-consistent regression’’ (SCR). As recommended in [40], the maximum likelihood method can be used to find the best values of parameters v:

$$\boldsymbol{a} = \arg\max p(\boldsymbol{y}|\boldsymbol{X}, \boldsymbol{v}) \quad (17)$$

In cases where $p(\boldsymbol{y}|\boldsymbol{X},\boldsymbol{a})$ and $p(\boldsymbol{a}|\boldsymbol{v})$ are the normal densities from equations (13)–(17), the following equation is derived:

$$\upsilon_k (a_k^2 + a^2 t_k) = 1, k = 1,...,m, \quad (18)$$

where t_k is the kth diagonal element of matrix T.

Due to their complex multidimensional nonlinear character, equation (18) can only be solved by iteration methods. Unlike the stepwise regression and other methods of combinatorial search, the SCR model includes all regressors. Nevertheless, the final model may contain several regressors truly describing the existing relationship. They can be easily identified based on their significance, which can be presented by the effective dimension of the regressor:

$$d_k (1 - \sigma^2 v_k t_k) = 1, k = 1,...,m, \quad (19)$$

Only those meeting a certain criterion, e.g. $d_k > 10^{-2}$, are left in the model.

The assumption of normality for *p(**y**|**X**,**a**)* and *p(**a**|**v**)* is not as restricted as seems to be the case. Normal distribution has an extreme property: it has the highest entropy for distributions with equal dispersion, and, in this sense, it is the ''worst'' among all possible distributions. Therefore, a solution obtained under the assumption of normality is rougher than it is theoretically possible for an exact residual distribution, but it is more robust, which is essential for the predictive power of a regression model. The regularized least-squares method (6) can be applied directly, without any statistical paradigm. However, the above-discussed statistical approach offers a useful tool for the optimisation of parameters v.

If residuals' dispersion σ^2 is unknown, then the following estimate s 2 can be used:

$$s^2 = \frac{\sum_i y_i (y_i - \sum_k x_{ik} a_k)}{(n - d)}, d = 1 + \sum_k d_k \tag{20}$$

Based on the above-described theory, we have developed an efficient SCR algorithm.

It is based on a modified Gram–Schmidt orthogonalization, which does not require the explicit inversion of a high-dimension matrix [40].

For a test molecule, the value of y can be calculated as follows:

$$y = \sum_k x_k a_k, k = 1,...,m \tag{21}$$

where m is the number of regressors (qQNA) left in the equation after the SCR-part of the training procedure.

The second method used implemented in the GUSAR 2019 program for selecting the optimal number of descriptors is the interpolation method for radial basis functions RBF [40]. The authors of the GUSAR 2019 program reports [40] that, unlike the RBF network, this method uses each input variable as a center of gravity. The learning process is performed on all input variables of the training set. As can be seen from equation (22), the approximating function y(x) in the case of the RBF interpolation is represented as the sum of *N* radial basis functions, each of which is related to another center x_i and weighted by the corresponding coefficient w_i.

$$y(x) = \sum_{i=1}^{N} w_i \varphi(\|x - x_i\|) = \Phi w \tag{22}$$

If the points x_i are different then the interpolation matrix Φ in the above equation is nonsingular. The weights w are calculated as:

$$w = \Phi^{-1} y \tag{23}$$

Assessing the weights is based on the simple least squares method [40].

The RBF-SCR method is the third tool of the GUSAR 2019 program for selecting the optimal number of descriptors. It has a 3-step algorithm:

1) selecting descriptors using the SCF method;
2) calculating the radial basis functions using the weighted coefficient of SCR as a criterion of similarity;
3) calculating the weighting coefficients RBF by the least squares.

The RBF-SCR method can be expressed as [40]:

$$y(x) = \sum_{i=1}^{N} w_i \varphi(\|ax - a_i x_i\|) = \Phi w, \quad (24)$$

where a is taken from equation (15). Weights a_i are a new elements as compared to equation (22).

The RBF and RBF-SCR interpolation is based on a linear radial basis function that allows modeling a variety of training sets with a high level of dissimilarity between the objects.

Additionally, the GUSAR program allows visualizing the contribution of each atom into the predicted value [20–22,40–43]. This capability is implemented in the QSAR models based on the QNA descriptors and, accordingly, in the consensus combination of the QSAR models designed in different modes. It opens opportunities to identify "strong" and "weak" points in the biologically active molecules and, consequently, to rationalize the conclusions about the replacement of certain fragments upon molecular design directed to enhancing/weakening the target property.

2.3. CONSTRUCTING QSAR MODELS

The QSAR models were designed in the GUSAR 2019 program as follows. To describe the structures of compounds within the program, two types of atom-centered descriptors were used, *viz.* substructural MNA, electrotopological QNA, and, additionally, three descriptors of the whole molecule (topological length, topological volume, and lipophilicity).

Self-consistent regression was used as a mathematical algorithm [20–22,40–43]. Previously, it has been shown [43] that self-consistent regression (SCR) can be successfully used to generate models from a large number of descriptors under different noise levels in the data. This method is correctly applied to modeling compounds with a rather high degree of similarity. Two other methods of selecting the optimal number of descriptors demonstrate good results when searching for quantitative structure–activity relationships in a series of structurally

dissimilar compounds. As the TYMS inhibitors are structurally similar, the RBF and RBF-SCF methods were not used in the present work.

The descriptors were automatically calculated from the structural formulas of chemical compounds, taking into account the valence and partial charges of the atoms. The optimal set of the descriptors for constructing particular regression equations was automatically selected by the self-consistent regression [47] and sliding control procedures [20–22,40–63]. The GUSAR 2019 program allows constructing both private regression dependencies and consensus models based on them. In this study, we use the consensus approach to construct the QSAR models. This allows reducing the variability of the predictions. Consensus models were designed in GUSAR 2019 automatically based on the principle of common similarity of particular regression dependencies [20–22,55–63].

Note that each of these partial models involved by the consensus model was made independently based on either QNA or MNA descriptors. As a result, 12 consensus QSAR models were designed. These models included 360 partial models. However, not all of them had acceptable statistical parameters. To select the most predictive models, a 20-fold crosscheck was performed for each model. These models have the R^2 values exceed 0.6 (from the cross-validation procedure after the randomized rejection of 20% of the training set). Each of the final consensus models M1–M2, M4–M5, M7–M8, M10–M11 is made up with 20 particular regression dependencies. Consensus models M3, M6, M9 and M12 include 320 regression equations. However, as the QNA and MNA descriptors have no direct physical meaning, the regression equations constructed on their basis are not explicitly displayed in the GUSAR 2019 program. Only the QSAR models satisfying the abovementioned condition have been further used for numerical predicting pIC_{50} for the compounds of the external training set.

2.4. ASSESSMENT OF THE RANGE OF APPLICABILITY

To assess the applicability of models, GUSAR 2019 provides three different approaches based on similarity, leverage, and accuracy previously described in detail [40,43].

Similarity. Using the Pearson correlation coefficients for each compound, we calculated the distances toward its nearest neighbors in the training set in the space of independent variables obtained after SCR. The compound is considered in the range of the model's applicability if the average value of these three distances is lower or equal to 0.7.

Leverage. The calculation of leverage allows estimating the contribution of each molecule to its own predicted value [40-45]:

$$\text{Leverage} = x^T\left(\mathbf{X}^\mathrm{T}\mathbf{X}\right)^{-1}x, \tag{25}$$

where x is the vector of descriptors of the tested compound and **X** is the matrix made up with rows corresponding to the descriptors of all the molecules of the training set [40]. The compound is considered out of the applicability range if its leverage is larger than 99 % in the distribution of the leverage values of the training set.

Accuracy degree (AD). Here, the prediction of the applicability range for each compound is calculated based on the prediction error for the three most similar compounds in the test set relative to the training set as a whole [40–43]:

$$\text{AD}_{value} = \text{RMSE}_{3NN} / \text{RMSE}_{train} \tag{26}$$

In the present study, a threshold value of 1 was used for AD.

3. RESULTS

Table S2. The validation parameters of the QSAR models estimated using the Xternal Validation Plus 1.2 program based on the experimental and predicted values of the HSV-1 TK inhibitors from test set TS1; $\Delta pIC_{50(TrS1)}$ = $\Delta pIC_{50(TrS3)}$ = 5.867374; $\Delta pIC_{50(TS1)}$ = 5.522879.

Comments	Code of models / Prediction parameters	QSAR model used for predicting pIC_{50}					
		M1	M2	M3	M7	M8	M9
Classical Metrics (100% data)	R^2	0.9323	0.9297	0.9385	0.9265	0.9125	0.9282
	R^2_0	0.9314	0.9286	0.9361	0.9265	0.9106	0.9252
	R'^2_0	0.9223	0.9181	0.9251	0.9223	0.8936	0.9101
	Q^2_{F1}	0.9377	0.9347	0.9420	0.9330	0.9179	0.9313
	Q^2_{F2}	0.9311	0.9278	0.9358	0.9259	0.9092	0.9240
	$\overline{R^2_m}$	0.8859	0.8628	0.8640	0.8986	0.8273	0.8380
	ΔR^2_m	0.0538	0.0580	0.0525	0.0261	0.0732	0.0626
	CCC	0.9632	0.9616	0.9655	0.9616	0.9510	0.9588
Classical Metrics (after removing 5% data with high residuals)	R^2	0.9486	0.9425	0.9544	0.9477	0.9339	0.9439
	R^2_0	0.9460	0.9413	0.9518	0.9473	0.9291	0.9405
	R'^2_0	0.8474	0.8529	0.8573	0.8790	0.7979	0.8298
	Q^2_{F1}	0.9510	0.9463	0.9556	0.9521	0.9316	0.9456
	Q^2_{F2}	0.9460	0.9413	0.9514	0.9472	0.9246	0.9405
	$\overline{R^2_m}$	0.8788	0.8903	0.8954	0.9067	0.8207	0.8687
	ΔR^2_m	0.0437	0.0464	0.0383	0.0414	0.0620	0.0481
	CCC	0.9710	0.9687	0.9738	0.9725	0.9591	0.9676
Mean absolute error and standard deviation for test set (100% data)	RMSEP	0.4418	0.4522	0.4264	0.4581	0.5071	0.4641
	MAE	0.3841	0.3914	0.3673	0.3823	0.4372	0.3987
	S.D.	0.2260	0.2344	0.2241	0.2612	0.2659	0.2458
	MAE+3×S.D.	1.0621	1.0946	1.0396	1.1659	1.2349	1.1361
	RMSEP	0.4049	0.4220	0.3836	0.4001	0.4783	0.4245

Mean absolute error and standard deviation for test set (after removing 5% data with high residuals)	MAE	0.3547	0.3652	0.3353	0.3416	0.4107	0.3671
	S.D.	0.2026	0.2193	0.1935	0.2162	0.2544	0.2212
	MAE+3×S.D.	0.9626	1.0231	0.9158	0.9903	1.1740	1.0308
Distribution of prediction errors (in %)	ωN in range $0.10\times\Delta IC_{50(TrS)}$	20.0000[a]	26.6667[a]	13.3333[a]	13.3333[b]	40.0000[b]	26.6667[b]
	ωN in range $0.15\times\Delta IC_{50(TrS)}$	0.0000[a]	0.0000[a]	0.0000[a]	0.0000[b]	0.0000[b]	0.0000[b]
	ωN in range $0.20\times\Delta IC_{50(TrS)}$	0.0000[a]	0.0000[a]	0.0000[a]	0.0000[b]	0.0000[b]	0.0000[b]
	ωN in range $0.25\times\Delta IC_{50(TrS)}$	0.0000[a]	0.0000[a]	0.0000[a]	0.0000[b]	0.0000[b]	0.0000[b]
Prediction quality	-	Good				Moderate	Good
Systematic error presence	-	Absent					

where R^2, R^2_0, and R'^2 are determination coefficients calculated with and without taking into account the origin;

$\overline{R^2_m}$ is the averaged determination coefficient of the regression function, calculated using values of determination coefficients on the ordinate axis (R^2_m) and using them on the abscissa (R'^2_m) respectively;

ΔR^2_m is the difference between R^2_m and R'^2_m;

Q^2_{F1} and $Q^2_{F2,}$ are determination coefficients calculated for the compounds of test set TS1 taking into account the average pIC_{50} value of the compounds from training and test sets, respectively;

CCC is the concordance correlation coefficient;

MAE is the mean absolute error;

S.D. is the standard deviation;

ωN is the percentage of test set TS1, for which the prediction error is less than the interval proportional to 0.1, 0.15, 0.20, and 0.25 of ΔpIC_{50} of training sets TrS1 (a) and TrS3 (b).

Table S3. The validation parameters of the QSAR models estimated using the Xternal Validation Plus 1.2 program based on the experimental and predicted of the HSV-2 TK inhibitors from internal test set TS2; $\Delta pIC_{50(TrS2)} = \Delta pIC_{50(TrS4)} = 6.24988$; $\Delta pIC_{50(TS1)} = 5.657577$.

Comments	Code of models / Prediction parameters	QSPR model used for predicting pIC_{50}					
		M4	M5	M6	M10	M11	M12
Classical Metrics (100% data)	R^2	0.9235	0.8897	0.8912	0.9038	0.8862	0.8834
	R^2_0	0.9189	0.8875	0.8906	0.9018	0.8858	0.8831
	$R^{2'}_0$	0.8993	0.8856	0.8718	0.8816	0.8775	0.8638
	Q^2_{F1}	0.9075	0.8806	0.8857	0.9015	0.8831	0.8807
	Q^2_{F2}	0.9073	0.8803	0.8854	0.9012	0.8827	0.8803

	$\overline{R_m^2}$	0.8683	0.8485	0.8496	0.8346	0.8439	0.8389
	ΔR^2_m	0.0620	0.0510	0.0792	0.0780	0.0140	0.0854
	CCC	0.9475	0.9384	0.9370	0.9455	0.9384	0.9347
Classical Metrics (after removing 5% data with high residuals)	R^2	0.9395	0.9324	0.9334	0.9317	0.9401	0.9416
	R^2_0	0.9390	0.9320	0.9309	0.9283	0.9399	0.9384
	R'^2_0	0.8591	0.8899	0.8186	0.8059	0.8703	0.8274
	Q^2_{F1}	0.9166	0.9115	0.9146	0.9236	0.9249	0.9238
	Q^2_{F2}	0.9164	0.9113	0.9145	0.9234	0.9247	0.9236
	$\overline{R_m^2}$	0.9164	0.8884	0.9076	0.8756	0.9171	0.9108
	ΔR^2_m	0.0310	0.0527	0.0515	0.0555	0.0175	0.0460
	CCC	0.9549	0.9538	0.9524	0.9574	0.9597	0.9575
Mean absolute error and standard deviation for test set (100% data)	RMSEP	0.5505	0.6255	0.6120	0.5682	0.6191	0.6254
	MAE	0.4690	0.4984	0.5039	0.4692	0.4766	0.5068
	S.D.	0.2983	0.3912	0.3595	0.3317	0.4090	0.3793
	MAE+3×S.D.	1.3639	1.672	1.5824	1.4643	1.7036	1.6447
Mean absolute error and standard deviation for test set (after removing 5% data with high residuals)	RMSEP	0.5020	0.5573	0.5472	0.5178	0.5133	0.5170
	MAE	0.4305	0.4460	0.4546	0.4282	0.4081	0.4388
	S.D.	0.2680	0.3469	0.3161	0.3022	0.3230	0.2836
	MAE+3×S.D.	1.2345	1.4866	1.4030	1.3349	1.3771	1.2898
Distribution of prediction errors (in %)	ωN in range $0.10\times\Delta IC_{50(TrS)}$	33.3333[a]	33.3333[a]	26.6667[a]	33.3333[b]	33.3333[b]	26.6667[b]
	ωN in range $0.15\times\Delta IC_{50(TrS)}$	6.6667[a]	20.0000[a]	13.3333[a]	13.3333[b]	13.3333[b]	13.3333[b]
	ωN in range $0.20\times\Delta IC_{50(TrS)}$	0.0000[a]	0.0000[a]	0.0000[a]	0.0000[b]	6.6667[b]	6.6667[b]
	ωN in range $0.25\times\Delta IC_{50(TrS)}$	0.0000[a]	0.0000[a]	0.0000[a]	0.0000[b]	0.0000[b]	0.0000[b]
Prediction quality	-	Good	Moderate				
Systematic error presence	-	Absent					

where R^2, R^2_0, and R'^2 are determination coefficients calculated with and without taking into account the origin;

$\overline{R_m^2}$ is the averaged determination coefficient of the regression function, calculated using values of determination coefficients on the ordinate axis (R^2_m) and using them on the abscissa (R'^2_m) respectively;

ΔR^2_m is the difference between R^2_m and R'^2_m;

Q^2_{F1} and Q^2_{F2}, are determination coefficients calculated for the compounds of test set TS2 taking into account the average pIC_{50} value of the compounds from training and test sets, respectively;

CCC is the concordance correlation coefficient;

MAE is the mean absolute error;

S.D. is the standard deviation;

ωN is the percentage of test set TS2, for which the prediction error is less than the interval proportional to 0.1, 0.15, 0.20, and 0.25 of ΔpIC_{50} of training sets TrS2 and TrS4 (b).

Table S4. The validation parameters of the QSAR models estimated using the Xternal Validation Plus 1.2 program based on the experimental and predicted of the HSV-1 TK inhibitors from test set TS3; $\Delta pIC_{50(TrS3)}$ = 5.867374; $\Delta pIC_{50(TS3)}$ = 4.80967.

Comments	Code of models / Prediction parameters	QSAR model used for predicting pIC_{50}		
		M7	M8	M9
Classical Metrics (100% data)	R^2	0.9461	0.9024	0.9330
	R^2_0	0.9450	0.9016	0.9323
	$R^{2'}_0$	0.9380	0.8976	0.9238
	Q^2_{F1}	0.9504	0.9103	0.9375
	Q^2_{F2}	0.9431	0.8971	0.9283
	$\overline{R^2_m}$	0.8790	0.8657	0.8604
	ΔR^2_m	0.0450	0.0727	0.0553
	CCC	0.9702	0.9483	0.9626
Classical Metrics (after removing 5% data with high residuals)	R^2	0.9566	0.9273	0.9394
	R^2_0	0.9566	0.9203	0.9391
	$R^{2'}_0$	0.9080	0.9182	0.8989
	Q^2_{F1}	0.9600	0.9253	0.9423
	Q^2_{F2}	0.9564	0.9196	0.9378
	$\overline{R^2_m}$	0.9283	0.8945	0.9161
	ΔR^2_m	0.0345	0.0550	0.0449
	CCC	0.9776	0.9618	0.9687
Mean absolute error and standard deviation for test set (100% data)	RMSEP	0.3720	0.5002	0.4175
	MAE	0.3225	0.3807	0.3227
	S.D.	0.1935	0.3389	0.2767
	MAE+3×S.D.	0.903	1.3974	1.1528
Mean absolute error and standard deviation for test set (after removing 5% data with high residuals)	RMSEP	0.3281	0.4273	0.3758
	MAE	0.2891	0.3247	0.2854
	S.D.	0.1626	0.2914	0.2564
	MAE+3×S.D.	0.7769	1.1987	1.0546
Distribution of prediction errors (in %)	ωN in range $0.10\times\Delta IC_{50(TrS)}$	8.3333	33.3333	33.3333
	ωN in range $0.15\times\Delta IC_{50(TrS)}$	0.0000	8.3333	0.0000
	ωN in range $0.20\times\Delta IC_{50(TrS)}$	0.0000	0.0000	0.0000
	ωN in range $0.25\times\Delta IC_{50(TrS)}$	0.0000	0.0000	0.0000
Prediction quality		Good	Moderate	Good
Systematic error presence	-	Absent		

where R^2, R^2_0, and R'^2 are determination coefficients calculated with and without taking into account the origin;

$\overline{R^2_m}$ is the averaged determination coefficient of the regression function, calculated using values of determination coefficients on the ordinate axis (R^2_m) and using them on the abscissa (R'^2_m) respectively;

ΔR^2_m is the difference between R^2_m and R'^2_m;

Q^2_{F1} and $Q^2_{F2,}$ are determination coefficients calculated for the compounds of test set TS1 taking into account the average pIC_{50} value of the compounds from training and test sets, respectively;

CCC is the concordance correlation coefficient;

MAE is the mean absolute error;

S.D. is the standard deviation;

ωN is the percentage of test set TS3, for which the prediction error is less than the interval proportional to 0.1, 0.15, 0.20, and 0.25 of ΔpIC_{50} of training sets TrS3.

Table S5. The validation parameters of the QSAR models estimated using the Xternal Validation Plus 1.2 program based ion the experimental and predicted of the HSV-2 TK inhibitors from internal test set TS4; $\Delta pIC_{50(TrS4)} = 6.24988$; $\Delta pIC_{50(TS4)} = 5.43573$.

Comments	Code of models / Prediction parameters	QSAR model used for predicting pIC_{50}		
		M10	M11	M12
Classical Metrics (100% data)	R^2	0.9016	0.9228	0.9130
	R^2_0	0.8978	0.9152	0.8994
	$R^{2'}_0$	0.8716	0.8898	0.8588
	Q^2_{F1}	0.8901	0.9148	0.8986
	Q^2_{F2}	0.8901	0.9148	0.8986
	$\overline{R^2_m}$	0.7785	0.8183	0.7587
	ΔR^2_m	0.0897	0.0700	0.0898
	CCC	0.9401	0.9515	0.9411
Classical Metrics (after removing 5% data with high residuals)	R^2	0.9276	0.9490	0.9370
	R^2_0	0.9276	0.9480	0.9332
	$R^{2'}_0$	0.8578	0.8688	0.8121
	Q^2_{F1}	0.9270	0.9435	0.9337
	Q^2_{F2}	0.9258	0.9426	0.9327
	$\overline{R^2_m}$	0.8825	0.9294	0.8664
	ΔR^2_m	0.0565	0.0335	0.0525
	CCC	0.9618	0.9693	0.9629
Mean absolute error and standard deviation for test set (100% data)	RMSEP	0.5746	0.5060	0.5521
	MAE	0.4423	0.3791	0.4149
	S.D.	0.3832	0.3500	0.3804
	MAE+3×S.D.	1.5919	1.4291	1.5561
Mean absolute error and standard deviation for test set (after removing 5% data with high residuals)	RMSEP	0.4486	0.3946	0.4275
	MAE	0.3623	0.3076	0.3359
	S.D.	0.2776	0.2592	0.2773
	MAE+3×S.D.	1.1949	1.0852	1.1678
Distribution of prediction errors (in %)	ωN in range $0.10\times\Delta IC_{50(TrS)}$	25.0000	16.6667	33.3333
	ωN in range $0.15\times\Delta IC_{50(TrS)}$	16.6667	8.3333	8.3333
	ωN in range $0.20\times\Delta IC_{50(TrS)}$	8.3333	0.0000	8.3333

	ωN in range $0.25\times\Delta IC_{50(TrS)}$	0.0000	0.0000	0.0000
Prediction quality		Good		
Systematic error presence	-	Absent		

where R^2, R^2_0, and R'^2 are determination coefficients calculated with and without taking into account the origin;

$\overline{R^2_m}$ is the averaged determination coefficient of the regression function, calculated using values of determination coefficients on the ordinate axis (R^2_m) and using them on the abscissa (R'^2_m) respectively;

ΔR^2_m is the difference between R^2_m and R'^2_m;

Q^2_{F1} and Q^2_{F2}, are determination coefficients calculated for the compounds of test set TS1 taking into account the average pIC_{50} value of the compounds from training and test sets, respectively;

CCC is the concordance correlation coefficient;

MAE is the mean absolute error;

S.D. is the standard deviation;

ωN is the percentage of test set TS4, for which the prediction error is less than the interval proportional to 0.1, 0.15, 0.20, and 0.25 of ΔpIC_{50} of training sets TrS4.

Table S6. Prediction of the pIC_{50} values for the TrS1 compounds using models M1–M3.*

Name in ZINC	pIC_{50}^{obs}	M1		M2		M3	
		pIC_{50}^{pred}	$\lvert\Delta pIC_{50}\rvert$	pIC_{50}^{pred}	$\lvert\Delta pIC_{50}\rvert$	pIC_{50}^{pred}	$\lvert\Delta pIC_{50}\rvert$
ZINC29402006	9.7212	9.4327	0.2885	9.1988	0.5224	9.1409	0.5803
ZINC29394944	9.5686	9.2100	0.3586	9.3619	0.2067	9.1904	0.3782
ZINC29391502	9.1805	8.1931	0.9874	8.8193	0.3612	8.7243	0.4562
ZINC29399678	9.0706	8.7943	0.2763	8.9225	0.1481	8.8952	0.1754
ZINC29397093	9.0315	8.8559	0.1756	9.0142	0.0173	8.8948	0.1367
ZINC29396056	9.0223	8.6031	0.4192	8.5058	0.5165	8.5342	0.4881
ZINC03777510	9.0000	8.1954	0.8046	8.0982	0.9018	8.0912	0.9088
ZINC29399482	8.8239	8.6353	0.1886	8.9218	0.0979	8.6858	0.1381
ZINC29401615	8.8239	8.7957	0.0282	9.0570	0.2331	8.8551	0.0312
ZINC29397210	8.7959	8.6646	0.1313	8.8176	0.0217	8.7461	0.0498
ZINC29393614	8.7696	8.6243	0.1453	8.7700	0.0004	8.7436	0.0260
ZINC03842454	8.7447	8.2599	0.4848	8.5505	0.1942	8.6199	0.1248
ZINC29402467	8.6576	8.7199	0.0623	8.8282	0.1706	8.6827	0.0251
ZINC29399940	8.5376	8.1777	0.3599	8.1138	0.4238	8.1340	0.4036
ZINC29401607	8.1549	7.9555	0.1994	7.8480	0.3069	7.7942	0.3607
ZINC29401248	8.0706	8.3222	0.2516	8.6255	0.5549	8.4512	0.3806
ZINC29396065	7.9393	8.1446	0.2053	8.0575	0.1182	8.0042	0.0649
ZINC28108646	7.5528	7.7063	0.1535	7.5550	0.0022	7.5374	0.0154
ZINC29402995	7.3979	7.0784	0.3195	7.1055	0.2924	7.1276	0.2703
ZINC29399967	7.0000	6.7361	0.2639	6.9881	0.0119	7.0405	0.0405
ZINC29403154	6.6990	7.0259	0.3269	7.1498	0.4508	7.0607	0.3617
ZINC29403158	6.6198	6.9311	0.3113	6.9506	0.3308	7.0126	0.3928
ZINC29400053	6.5850	7.3092	0.7242	7.2010	0.6160	7.2158	0.6308
ZINC29403322	6.5229	6.8439	0.3210	6.9403	0.4174	6.9071	0.3842

ZINC29400764	6.1549	6.5771	0.4222	6.6015	0.4466	6.6277	0.4728
ZINC29396364	5.7825	6.6916	0.9091	6.4718	0.6893	6.4250	0.6425
ZINC05117141	6.8239	6.1397	0.6842	6.0687	0.7552	6.1865	0.6374
ZINC13602973	6.0000	5.4754	0.5246	5.4388	0.5612	5.4757	0.5243
ZINC05542645	5.8861	5.4100	0.4761	5.4160	0.4701	5.4591	0.4270
ZINC13726285	5.5229	5.5490	0.0261	5.6065	0.0836	5.4784	0.0445
ZINC13726289	5.4815	5.1286	0.3529	5.0880	0.3935	5.0580	0.4235
ZINC13726297	5.1549	5.1062	0.0487	5.2262	0.0713	5.1375	0.0174
ZINC17838260	5.0969	4.6954	0.4015	4.5833	0.5136	4.6734	0.4235
ZINC05542666	5.0706	5.0378	0.0328	5.0124	0.0582	4.9873	0.0833
ZINC26176052	5.0000	5.0249	0.0249	5.0766	0.0766	5.0240	0.0240
ZINC13726301	4.9208	5.1561	0.2353	5.0888	0.1680	5.1061	0.1853
ZINC13602985	4.8239	4.9032	0.0793	4.8316	0.0077	4.8979	0.0740
ZINC13726305	4.6990	4.6836	0.0154	4.7452	0.0462	4.7409	0.0419
ZINC01648710	4.6021	4.8031	0.2010	4.8369	0.2348	4.8614	0.2593
ZINC13726313	4.5229	4.8796	0.3567	4.8386	0.3157	4.8074	0.2845
ZINC13726320	4.3979	4.7509	0.3530	4.6074	0.2095	4.6669	0.2690
ZINC26161976	4.3468	4.5677	0.2209	4.5703	0.2235	4.5988	0.2520
ZINC13602968	4.3010	4.6147	0.3137	4.4836	0.1826	4.6110	0.3100
ZINC05114684	4.3010	4.9723	0.6713	4.8264	0.5254	4.8584	0.5574
ZINC26165821	4.2596	4.7048	0.4452	4.6600	0.4004	4.6384	0.3788
ZINC13756664	4.2596	4.5440	0.2844	4.5335	0.2739	4.5042	0.2446
ZINC13602961	4.0000	4.7173	0.7173	4.5943	0.5943	4.4934	0.4934
ZINC13602976	3.8539	4.4308	0.5769	4.3648	0.5109	4.3755	0.5216
ZINC13644900	6.8239	6.3025	0.5214	6.3308	0.4931	6.2948	0.5291
ZINC13644918	6.4559	6.1599	0.2960	5.9702	0.4857	6.0702	0.3857
ZINC14977328	6.3010	5.9355	0.3655	5.9410	0.3600	5.9909	0.3101
ZINC13644950	6.2596	6.3987	0.1391	6.0917	0.1679	6.1520	0.1076
ZINC14977331	6.1249	5.8079	0.3170	5.7074	0.4175	5.7883	0.3366
ZINC13644892	6.1135	5.9009	0.2126	6.1213	0.0078	5.8859	0.2276
ZINC13644863	6.0000	5.6887	0.3113	5.4945	0.5055	5.6241	0.3759
ZINC22933690	6.0000	5.7673	0.2327	5.8389	0.1611	5.8630	0.1370
ZINC01540304	5.8861	5.3924	0.4937	5.3421	0.5440	5.3947	0.4914
ZINC13644852	5.6990	5.3747	0.3243	5.3504	0.3486	5.3943	0.3047
ZINC14977337	5.6990	6.0979	0.3989	6.0423	0.3433	6.0802	0.3812
ZINC13644880	5.6778	5.7551	0.0773	5.6836	0.0058	5.7114	0.0336
ZINC13644937	5.6383	5.4907	0.1476	5.5143	0.1240	5.5730	0.0653
ZINC13644943	5.6021	5.7939	0.1918	5.8263	0.2242	5.7685	0.1664
ZINC13644867	5.4437	5.3749	0.0688	5.3230	0.1207	5.3373	0.1064
ZINC13644849	5.3565	5.1937	0.1628	5.1740	0.1825	5.3077	0.0488
ZINC13644873	5.3279	5.4397	0.1118	5.4290	0.1011	5.4139	0.0860
ZINC13644877	5.2840	5.5170	0.2330	5.3738	0.0898	5.4069	0.1229
ZINC13644934	5.2441	5.4516	0.2075	5.4758	0.2317	5.4120	0.1679
ZINC22933683	5.1487	5.5892	0.4405	5.6528	0.5041	5.4666	0.3179
ZINC13644928	5.0862	5.3331	0.2469	5.2124	0.1262	5.2735	0.1873
ZINC13644854	4.8239	5.2119	0.3880	5.2275	0.4036	5.3075	0.4836

ZINC14977334	4.7959	5.2945	0.4986	5.2508	0.4549	5.2675	0.4716
ZINC13644886	4.7825	5.4522	0.6697	5.4981	0.7156	5.4580	0.6755
ZINC06119296	4.2218	4.5331	0.3113	4.6419	0.4201	4.5940	0.3722

* The falling out results are marked by red.

Table S7. Prediction of the pIC_{50} values for the TrS2 compounds using models M4–M6.*

Name in ZINC	pIC_{50}^{obs}	M4		M5		M6	
		pIC_{50}^{pred}	$\|\Delta pIC_{50}\|$	pIC_{50}^{pred}	$\|\Delta pIC_{50}\|$	pIC_{50}^{pred}	$\|\Delta pIC_{50}\|$
ZINC29394944	10.0458	9.9831	0.0627	10.0843	0.0385	9.8265	0.2193
ZINC29398797	10.0000	9.9212	0.0788	9.7769	0.2231	9.8088	0.1912
ZINC29402006	9.9586	10.0519	0.0933	9.7626	0.1960	9.6722	0.2864
ZINC29390641	9.9586	9.4660	0.4926	9.4622	0.4964	9.5353	0.4233
ZINC29399940	9.8861	9.3409	0.5452	9.2142	0.6719	9.2259	0.6602
ZINC29396056	9.8861	9.4207	0.4654	9.4075	0.4786	9.3308	0.5553
ZINC29399678	9.8539	9.5844	0.2695	9.8196	0.0343	9.7669	0.0870
ZINC29399950	9.8539	9.7355	0.1184	9.8845	0.0306	9.7098	0.1441
ZINC29397210	9.7212	9.4223	0.2989	9.7000	0.0212	9.5916	0.1296
ZINC03842454	9.7212	9.1828	0.5384	9.4732	0.2480	9.3214	0.3998
ZINC29401615	9.6778	9.6538	0.0240	9.8718	0.1940	9.6456	0.0322
ZINC29393614	9.6778	9.6233	0.0545	9.7491	0.0713	9.6788	0.0010
ZINC29402467	9.3665	9.5323	0.1658	9.5876	0.2211	9.5034	0.1369
ZINC29401248	9.1549	9.2069	0.0520	9.3421	0.1872	9.2703	0.1154
ZINC29401607	8.9208	8.7706	0.1502	8.5556	0.3652	8.5300	0.3908
ZINC29396065	8.8539	9.0396	0.1857	8.7594	0.0945	8.7987	0.0552
ZINC29399935	8.5850	8.9549	0.3699	8.7533	0.1683	8.6625	0.0775
ZINC28108646	8.2840	8.2787	0.0053	8.2007	0.0833	8.2059	0.0781
ZINC29402995	8.0757	7.3969	0.6788	7.5119	0.5638	7.4453	0.6304
ZINC29399967	7.5229	7.2079	0.3150	7.3387	0.1842	7.3827	0.1402
ZINC29400053	7.3565	8.0407	0.6842	7.9204	0.5639	7.9855	0.6290
ZINC29400479	7.2218	7.1668	0.0550	7.3781	0.1563	7.3327	0.1109
ZINC29403322	7.1549	7.2877	0.1328	7.3764	0.2215	7.3783	0.2234
ZINC29403154	7.0458	7.2926	0.2468	7.3721	0.3263	7.3615	0.3157
ZINC29403158	7.0458	7.7473	0.7015	7.2935	0.2477	7.4937	0.4479
ZINC29400764	6.8539	6.9662	0.1123	7.2826	0.4287	7.1880	0.3341
ZINC29394218	6.5229	6.8640	0.3411	6.8884	0.3655	6.9624	0.4395
ZINC29396364	6.3768	7.1634	0.7866	6.9726	0.5958	7.0519	0.6751
ZINC05117141	7.0000	6.3119	0.6881	6.3300	0.6700	6.3877	0.6123
ZINC05542645	6.4559	5.9904	0.4655	5.9295	0.5264	5.8647	0.5912
ZINC13602979	5.3979	5.1199	0.2780	5.2268	0.1711	5.2043	0.1936
ZINC13726289	5.5229	5.2733	0.2496	5.3921	0.1308	5.4178	0.1051
ZINC13726297	5.0969	5.3322	0.2353	5.4176	0.3207	5.4174	0.3205
ZINC05542904	5.6021	5.6069	0.0048	5.6350	0.0329	5.5843	0.0178
ZINC17838260	5.7959	5.4100	0.3859	5.3041	0.4918	5.3095	0.4864
ZINC05542666	5.2596	5.1769	0.0827	5.2373	0.0223	5.2411	0.0185
ZINC26176052	4.8539	5.2055	0.3516	5.2804	0.4265	5.2647	0.4108

ZINC13726301	5.6021	5.5964	0.0057	5.5151	0.0870	5.5846	0.0175
ZINC13602985	5.0000	5.2076	0.2076	5.2132	0.2132	5.2537	0.2537
ZINC13726305	4.6021	5.0044	0.4023	5.0624	0.4603	5.0736	0.4715
ZINC05542665	4.6990	4.5595	0.1395	4.7594	0.0604	4.6305	0.0685
ZINC01648710	4.6990	5.1355	0.4365	5.0324	0.3334	5.0931	0.3941
ZINC13726313	4.3979	4.8423	0.4444	4.9216	0.5237	4.8215	0.4236
ZINC13726320	5.0000	5.1562	0.1562	5.1795	0.1795	5.2115	0.2115
ZINC26161976	5.6990	5.2315	0.4675	5.2015	0.4975	5.2335	0.4655
ZINC13602982	5.6576	5.4997	0.1579	5.4363	0.2213	5.4707	0.1869
ZINC26165821	5.0969	5.2411	0.1442	5.1055	0.0086	5.2861	0.1892
ZINC13602961	4.5229	5.1388	0.6159	5.0254	0.5025	5.0473	0.5244
ZINC05542648	4.3979	4.6197	0.2218	4.5111	0.1132	4.5627	0.1648
ZINC26170064	5.0000	4.8475	0.1525	5.0704	0.0704	4.9331	0.0669
ZINC13644900	7.8861	7.2313	0.6548	7.3643	0.5218	7.4009	0.4852
ZINC13644892	7.1871	6.9358	0.2513	6.8848	0.3023	6.8387	0.3484
ZINC22933690	6.8861	6.8430	0.0431	6.9755	0.0894	7.0275	0.1414
ZINC13644937	6.8539	6.5173	0.3366	6.5674	0.2865	6.6540	0.1999
ZINC13644943	6.8239	6.6758	0.1481	6.8364	0.0125	6.7468	0.0771
ZINC13644953	6.6576	6.8348	0.1772	6.9155	0.2579	7.0643	0.4067
ZINC13644883	6.6576	6.3957	0.2619	6.2957	0.3619	6.3800	0.2776
ZINC13644870	6.6383	6.4502	0.1881	6.2905	0.3478	6.3535	0.2848
ZINC13644934	6.5086	6.4711	0.0375	6.4252	0.0834	6.4137	0.0949
ZINC13644873	6.4202	6.2635	0.1567	6.3213	0.0989	6.3242	0.0960
ZINC13644880	6.3768	6.4724	0.0956	6.5113	0.1345	6.4339	0.0571
ZINC01540304	6.3010	5.7990	0.5020	5.7492	0.5518	5.8652	0.4358
ZINC13644867	6.2076	6.0626	0.1450	6.1171	0.0905	6.1208	0.0868
ZINC13644863	6.1549	5.8604	0.2945	5.8209	0.3340	5.7882	0.3667
ZINC13644928	6.1487	6.4047	0.2560	6.2052	0.0565	6.3051	0.1564
ZINC22933683	6.0969	6.5660	0.4691	6.5189	0.4220	6.3710	0.2741
ZINC13644886	6.0000	6.6062	0.6062	6.6230	0.6230	6.6343	0.6343
ZINC13644852	5.8239	5.5544	0.2695	5.5445	0.2794	5.5525	0.2714
ZINC14977328	5.6990	5.8317	0.1327	5.4749	0.2241	5.6984	0.0006
ZINC14977331	5.0458	5.4560	0.4102	5.1376	0.0918	5.3143	0.2685
ZINC13644858	4.8539	5.1398	0.2859	5.3729	0.5190	5.2871	0.4332
ZINC14977334	4.4318	5.0846	0.6528	5.0087	0.5769	4.9453	0.5135
ZINC13644854	4.2676	4.8650	0.5974	4.6523	0.3847	4.6851	0.4175
ZINC06119296	3.7959	4.3479	0.5520	4.2974	0.5015	4.3144	0.5185

* The falling out results are marked by red.

Table S8. Prediction of the pIC_{50} values for the TrS3 compounds using models M7–M9.*

Name in ZINC	pIC_{50}^{obs}	M7		M8		M9	
		pIC_{50}^{pred}	$\lvert\Delta pIC_{50}\rvert$	pIC_{50}^{pred}	$\lvert\Delta pIC_{50}\rvert$	pIC_{50}^{pred}	$\lvert\Delta pIC_{50}\rvert$
ZINC29402006	9.7212	9.5567	0.1645	9.1390	0.5822	9.1393	0.5819
ZINC29394944	9.5686	9.1553	0.4133	9.3424	0.2262	9.1213	0.4473
ZINC29391502	9.1805	8.3287	0.8518	8.7742	0.4063	8.7310	0.4495
ZINC29399678	9.0706	8.7669	0.3037	8.9861	0.0845	8.8732	0.1974

ZINC29396056	9.0223	8.6643	0.3580	8.6084	0.4139	8.5991	0.4232
ZINC03777510	9.0000	8.1946	0.8054	8.1682	0.8318	8.2569	0.7431
ZINC29399482	8.8239	8.6003	0.2236	8.8715	0.0476	8.6869	0.1370
ZINC29401615	8.8239	8.8401	0.0162	9.0888	0.2649	8.8454	0.0215
ZINC29397210	8.7959	8.6583	0.1376	8.8203	0.0244	8.7407	0.0552
ZINC03842454	8.7447	8.4238	0.3209	8.6528	0.0919	8.6391	0.1056
ZINC29402467	8.6576	8.7620	0.1044	8.7809	0.1233	8.6856	0.0280
ZINC29399940	8.5376	8.2840	0.2536	8.1674	0.3702	8.1069	0.4307
ZINC29401607	8.1549	8.0293	0.1256	7.8398	0.3151	7.7712	0.3837
ZINC29401248	8.0706	8.4671	0.3965	8.5742	0.5036	8.4994	0.4288
ZINC28108646	7.5528	7.6365	0.0837	7.5554	0.0026	7.5343	0.0185
ZINC29402995	7.3979	7.0065	0.3914	7.1731	0.2248	7.1397	0.2582
ZINC29399967	7.0000	6.7995	0.2005	7.0835	0.0835	7.0849	0.0849
ZINC29403158	6.6198	6.9074	0.2876	6.9118	0.2920	7.0127	0.3929
ZINC29400053	6.5850	7.2565	0.6715	7.1888	0.6038	7.2724	0.6874
ZINC29403322	6.5229	6.8675	0.3446	6.9831	0.4602	6.9433	0.4204
ZINC29400764	6.1549	6.5712	0.4163	6.7582	0.6033	6.6646	0.5097
ZINC29396364	5.7825	6.6510	0.8685	6.4781	0.6956	6.4710	0.6885
ZINC05117141	6.8239	6.1483	0.6756	6.0750	0.7489	6.1348	0.6891
ZINC13602973	6.0000	5.5010	0.4990	5.4855	0.5145	5.4397	0.5603
ZINC05542645	5.8861	5.4292	0.4569	5.4870	0.3991	5.4142	0.4719
ZINC13726285	5.5229	5.5208	0.0021	5.5730	0.0501	5.4724	0.0505
ZINC13726289	5.4815	5.1124	0.3691	5.0196	0.4619	5.0564	0.4251
ZINC13726297	5.1549	5.0509	0.1040	5.2041	0.0492	5.1401	0.0148
ZINC05542666	5.0706	5.0131	0.0575	5.0434	0.0272	4.9858	0.0848
ZINC26176052	5.0000	4.9675	0.0325	5.1165	0.1165	5.0351	0.0351
ZINC13726301	4.9208	5.2508	0.3300	5.1002	0.1794	5.1242	0.2034
ZINC13602985	4.8239	4.8510	0.0271	4.8691	0.0452	4.8872	0.0633
ZINC13726305	4.6990	4.5764	0.1226	4.6895	0.0095	4.7404	0.0414
ZINC01648710	4.6021	4.8312	0.2291	4.8859	0.2838	4.8734	0.2713
ZINC13726313	4.5229	4.9251	0.4022	4.8273	0.3044	4.8560	0.3331
ZINC26161976	4.3468	4.4725	0.1257	4.4737	0.1269	4.5927	0.2459
ZINC13602968	4.3010	4.5512	0.2502	4.4844	0.1834	4.5844	0.2834
ZINC05114684	4.3010	4.8594	0.5584	4.8033	0.5023	4.8413	0.5403
ZINC26165821	4.2596	4.6751	0.4155	4.5780	0.3184	4.6447	0.3851
ZINC13756664	4.2596	4.6577	0.3981	4.5207	0.2611	4.5428	0.2832
ZINC13602961	4.0000	4.7310	0.7310	4.5911	0.5911	4.5016	0.5016
ZINC13602976	3.8539	4.3693	0.5154	4.2857	0.4318	4.3530	0.4991
ZINC13644900	6.8239	6.2634	0.5605	6.2785	0.5454	6.2439	0.5800
ZINC13644918	6.4559	6.0948	0.3611	5.9283	0.5276	6.0991	0.3568
ZINC14977328	6.3010	6.0090	0.2920	5.9881	0.3129	6.0417	0.2593
ZINC14977331	6.1249	5.8389	0.2860	5.7936	0.3313	5.8460	0.2789
ZINC13644892	6.1135	5.9549	0.1586	6.0762	0.0373	5.8950	0.2185
ZINC13644863	6.0000	5.6698	0.3302	5.5967	0.4033	5.6374	0.3626
ZINC01540304	5.8861	5.5854	0.3007	5.5205	0.3656	5.5376	0.3485
ZINC13644852	5.6990	5.4398	0.2592	5.4364	0.2626	5.4516	0.2474

ZINC14977337	5.6990	6.0653	0.3663	6.0717	0.3727	6.1142	0.4152
ZINC13644937	5.6383	5.4562	0.1821	5.4959	0.1424	5.5648	0.0735
ZINC13644943	5.6021	5.7321	0.1300	5.7638	0.1617	5.7573	0.1552
ZINC13644867	5.4437	5.4304	0.0133	5.3831	0.0606	5.3273	0.1164
ZINC13644873	5.3279	5.3841	0.0562	5.4718	0.1439	5.3839	0.0560
ZINC13644877	5.2840	5.5234	0.2394	5.4189	0.1349	5.4549	0.1709
ZINC13644934	5.2441	5.5231	0.2790	5.4521	0.2080	5.4174	0.1733
ZINC22933683	5.1487	5.6079	0.4592	5.6163	0.4676	5.4517	0.3030
ZINC13644928	5.0862	5.3857	0.2995	5.1893	0.1031	5.3014	0.2152
ZINC14977334	4.7959	5.2800	0.4841	5.4140	0.6181	5.3486	0.5527
ZINC13644886	4.7825	5.3093	0.5268	5.4188	0.6363	5.3631	0.5806

* The falling out results are marked by red.

Table S9. Prediction of the pIC_{50} values for the TrS4 compounds using models M10–M12.*

Name in ZINC	pIC_{50}^{obs}	M10		M11		M12	
		pIC_{50}^{pred}	$\|\Delta pIC_{50}\|$	pIC_{50}^{pred}	$\|\Delta pIC_{50}\|$	pIC_{50}^{pred}	$\|\Delta pIC_{50}\|$
ZINC29394944	10.0458	9.9978	0.0480	10.0842	0.0384	9.8903	0.1555
ZINC29398797	10.0000	9.9799	0.0201	9.8022	0.1978	9.8416	0.1584
ZINC29390641	9.9586	9.4616	0.4970	9.4311	0.5275	9.5078	0.4508
ZINC29399940	9.8861	9.3146	0.5715	9.1745	0.7116	9.3521	0.5340
ZINC29396056	9.8861	9.3751	0.5110	9.2668	0.6193	9.3087	0.5774
ZINC29399678	9.8539	9.6132	0.2407	9.8404	0.0135	9.7819	0.0720
ZINC29399950	9.8539	9.7651	0.0888	9.8503	0.0036	9.7100	0.1439
ZINC03842454	9.7212	9.3029	0.4183	9.5710	0.1502	9.4072	0.3140
ZINC29401615	9.6778	9.7031	0.0253	9.8647	0.1869	9.7066	0.0288
ZINC29393614	9.6778	9.6025	0.0753	9.7992	0.1214	9.7113	0.0335
ZINC29402467	9.3665	9.5258	0.1593	9.5545	0.1880	9.4086	0.0421
ZINC29401248	9.1549	9.1600	0.0051	9.3064	0.1515	9.2749	0.1200
ZINC29396065	8.8539	8.9599	0.1060	8.6467	0.2072	8.8049	0.0490
ZINC29399935	8.5850	8.8708	0.2858	8.6718	0.0868	8.6187	0.0337
ZINC28108646	8.2840	8.2116	0.0724	7.9931	0.2909	8.0976	0.1864
ZINC29402995	8.0757	7.3343	0.7414	7.5407	0.5350	7.4361	0.6396
ZINC29400053	7.3565	8.0601	0.7036	7.8826	0.5261	7.8750	0.5185
ZINC29400479	7.2218	7.1276	0.0942	7.3081	0.0863	7.3038	0.0820
ZINC29403322	7.1549	7.2123	0.0574	7.4662	0.3113	7.3564	0.2015
ZINC29403154	7.0458	7.3045	0.2587	7.3706	0.3248	7.3663	0.3205
ZINC29400764	6.8539	6.9584	0.1045	7.2441	0.3902	7.1582	0.3043
ZINC29394218	6.5229	6.8107	0.2878	6.9121	0.3892	6.9497	0.4268
ZINC29396364	6.3768	7.2220	0.8452	7.0336	0.6568	7.0634	0.6866
ZINC05117141	7.0000	6.3612	0.6388	6.3145	0.6855	6.4395	0.5605
ZINC05542645	6.4559	5.8431	0.6128	5.8936	0.5623	5.8469	0.6090
ZINC13602979	5.3979	5.1046	0.2933	5.2614	0.1365	5.2109	0.1870
ZINC13726289	5.5229	5.2316	0.2913	5.3639	0.1590	5.3646	0.1583
ZINC13726297	5.0969	5.2910	0.1941	5.4348	0.3379	5.3707	0.2738
ZINC05542904	5.6021	5.7158	0.1137	5.7063	0.1042	5.6865	0.0844

ZINC17838260	5.7959	5.4050	0.3909	5.3205	0.4754	5.3502	0.4457
ZINC26176052	4.8539	5.3112	0.4573	5.2616	0.4077	5.3588	0.5049
ZINC13726301	5.6021	5.6114	0.0093	5.6209	0.0188	5.6683	0.0662
ZINC13602985	5.0000	5.1519	0.1519	5.2831	0.2831	5.2411	0.2411
ZINC13726305	4.6021	5.0210	0.4189	5.0679	0.4658	5.0534	0.4513
ZINC05542665	4.6990	4.5078	0.1912	4.8164	0.1174	4.6354	0.0636
ZINC01648710	4.6990	5.1035	0.4045	5.0679	0.3689	5.1208	0.4218
ZINC13726313	4.3979	5.0404	0.6425	4.9724	0.5745	4.8291	0.4312
ZINC13726320	5.0000	5.2172	0.2172	5.1868	0.1868	5.2447	0.2447
ZINC26161976	5.6990	5.2507	0.4483	5.2335	0.4655	5.2311	0.4679
ZINC13602982	5.6576	5.6109	0.0467	5.4644	0.1932	5.5147	0.1429
ZINC26165821	5.0969	5.3123	0.2154	5.2811	0.1842	5.3001	0.2032
ZINC05542648	4.3979	4.6354	0.2375	4.5649	0.1670	4.6052	0.2073
ZINC13644900	7.8861	7.2431	0.6430	7.3058	0.5803	7.3021	0.5840
ZINC13644892	7.1871	6.9982	0.1889	6.9364	0.2507	6.8680	0.3191
ZINC22933690	6.8861	7.0672	0.1811	7.0106	0.1245	7.0602	0.1741
ZINC13644937	6.8539	6.7311	0.1228	6.6438	0.2101	6.7122	0.1417
ZINC13644943	6.8239	6.7416	0.0823	6.8484	0.0245	6.7672	0.0567
ZINC13644883	6.6576	6.4020	0.2556	6.2241	0.4335	6.3936	0.2640
ZINC13644870	6.6383	6.4259	0.2124	6.2721	0.3662	6.2950	0.3433
ZINC13644934	6.5086	6.5454	0.0368	6.5088	0.0002	6.4845	0.0241
ZINC13644880	6.3768	6.4282	0.0514	6.5318	0.1550	6.4981	0.1213
ZINC01540304	6.3010	5.8559	0.4451	5.7242	0.5768	5.8629	0.4381
ZINC13644867	6.2076	5.8840	0.3236	6.0413	0.1663	6.0597	0.1479
ZINC13644863	6.1549	5.8527	0.3022	5.6818	0.4731	5.7930	0.3619
ZINC22933683	6.0969	6.5111	0.4142	6.5305	0.4336	6.3679	0.2710
ZINC13644886	6.0000	6.7274	0.7274	6.6448	0.6448	6.6948	0.6948
ZINC13644852	5.8239	5.5706	0.2533	5.5275	0.2964	5.5668	0.2571
ZINC14977331	5.0458	5.4312	0.3854	5.1112	0.0654	5.2607	0.2149
ZINC13644858	4.8539	5.2012	0.3473	5.3223	0.4684	5.3054	0.4515
ZINC14977334	4.4318	5.0452	0.6134	4.7510	0.3192	4.9659	0.5341
ZINC13644854	4.2676	4.8725	0.6049	4.6396	0.3720	4.6897	0.4221
ZINC06119296	3.7959	4.3149	0.5190	4.2915	0.4956	4.2829	0.4870

* The falling out results are marked by red.

Table S10. Prediction of the pIC_{50} values for the TS1 compounds using models M1–M3, M7–M9.*

Name in ZINC	pIC_{50}^{obs}	M1		M2		M3		M7		M9		M10	
		pIC_{50}^{pred}	$\|\Delta pIC_{50}\|$	pIC_{50}^{pred}	$\|\Delta pIC_{50}\|$	pIC_{50}^{pred}	$\|\Delta pIC_{50}\|$	pIC_{50}^{pred}	$\|\Delta pIC_{50}\|$	pIC_{50}^{pred}	$\|\Delta pIC_{50}\|$	pIC_{50}^{pred}	$\|\Delta pIC_{50}\|$
ZINC29398797	9.5229	9.0725	0.4504	8.9849	0.5380	8.9459	0.5770	9.0502	0.4727	8.9612	0.5617	8.9447	0.5782
ZINC29399950	9.0000	8.8194	0.1806	9.0620	0.0620	8.8254	0.1746	9.0208	0.0208	9.0966	0.0966	8.8381	0.1619
ZINC29390641	8.6778	8.3716	0.3062	8.5326	0.1452	8.5693	0.1085	8.4192	0.2586	8.5336	0.1442	8.5535	0.1243
ZINC29399935	7.6383	8.1283	0.4900	8.0391	0.4008	7.8910	0.2527	8.1540	0.5157	7.8888	0.2505	7.9639	0.3256
ZINC29400479	6.6990	6.8165	0.1175	6.9720	0.2730	7.0652	0.3662	6.7352	0.0362	7.0650	0.3660	7.1090	0.4100
ZINC29394218	6.0000	6.4352	0.4352	6.7578	0.7578	6.8164	0.8164	6.4566	0.4566	6.7918	0.7918	6.8413	0.8413
ZINC13602979	5.6021	4.8254	0.7767	4.9478	0.6543	4.9510	0.6511	4.7978	0.8043	4.9697	0.6324	4.9332	0.6689
ZINC05542904	5.0969	5.0607	0.0362	5.3172	0.2203	5.2202	0.1233	4.9940	0.1029	5.4319	0.3350	5.2316	0.1347
ZINC05542665	4.6021	4.4305	0.1716	4.4657	0.1364	4.3630	0.2391	4.3810	0.2211	4.4729	0.1292	4.3989	0.2032
ZINC13602982	4.3010	4.9180	0.6170	4.8414	0.5404	4.8438	0.5428	4.5930	0.2920	4.9238	0.6228	4.8915	0.5905
ZINC05542648	4.0000	4.4926	0.4926	4.6016	0.6016	4.5313	0.5313	4.4970	0.4970	4.7509	0.7509	4.6717	0.6717
ZINC13644953	6.3872	5.5922	0.7950	5.7199	0.6673	6.1142	0.2730	5.4355	0.9517	5.5783	0.8089	6.1032	0.2840
ZINC13644883	5.7959	5.5020	0.2939	5.4348	0.3611	5.4208	0.3751	5.4348	0.3611	5.3851	0.4108	5.4123	0.3836
ZINC13644870	5.3468	5.6343	0.2875	5.3949	0.0481	5.3812	0.0344	5.5968	0.2500	5.3858	0.0390	5.3683	0.0215
ZINC13644858	4.9208	5.2316	0.3108	5.3857	0.4649	5.3654	0.4446	5.4144	0.4936	5.5396	0.6188	5.5020	0.5812

* The falling out results are marked by red.

Table S11. Prediction of the pIC_{50} values for the TS2 compounds using models M4–M6, M10–M12.*

Name in ZINC	pIC_{50}^{obs}	M1		M2		M3		M7		M9		M10	
		pIC_{50}^{pred}	$\|\Delta pIC_{50}\|$	pIC_{50}^{pred}	$\|\Delta pIC_{50}\|$	pIC_{50}^{pred}	$\|\Delta pIC_{50}\|$	pIC_{50}^{pred}	$\|\Delta pIC_{50}\|$	pIC_{50}^{pred}	$\|\Delta pIC_{50}\|$	pIC_{50}^{pred}	$\|\Delta pIC_{50}\|$
ZINC29397093	9.9586	9.6101	0.3485	9.7078	0.2508	9.4848	0.4738	9.5355	0.4231	9.6095	0.3491	9.5064	0.4522
ZINC29391502	9.8539	9.1001	0.7538	9.6915	0.1624	9.5315	0.3224	9.7416	0.1123	9.7668	0.0871	9.5945	0.2594
ZINC29399482	9.5528	9.0835	0.4693	9.5499	0.0029	9.2280	0.3248	9.0030	0.5498	9.4323	0.1205	9.1200	0.4328
ZINC03777510	8.4815	7.6097	0.8718	7.4288	1.0527	7.6399	0.8416	7.7106	0.7709	7.4353	1.0462	7.7290	0.7525
ZINC13602973	6.2218	5.3769	0.8449	5.2392	0.9826	5.2851	0.9367	5.5132	0.7086	5.4676	0.7542	5.3693	0.8525
ZINC13726285	5.8861	5.6987	0.1874	5.5830	0.3031	5.4308	0.4553	5.6988	0.1873	5.6583	0.2278	5.5358	0.3503
ZINC13602968	5.2218	5.0541	0.1677	5.0302	0.1916	5.1208	0.1010	5.0821	0.1397	5.1325	0.0893	5.1411	0.0807
ZINC05114684	4.3010	5.3095	1.0085	5.1963	0.8953	5.4201	1.1191	5.2995	0.9985	5.2036	0.9026	5.3515	1.0505
ZINC13756664	5.0000	4.7454	0.2546	4.7496	0.2504	4.8583	0.1417	4.7151	0.2849	4.8723	0.1277	4.7908	0.2092
ZINC13602976	4.6990	5.2066	0.5076	5.0348	0.3358	5.2420	0.5430	5.4952	0.7962	5.0802	0.3812	5.2676	0.5686
ZINC13644918	7.3279	6.9537	0.3742	6.5686	0.7593	6.8800	0.4479	7.1732	0.1547	6.6903	0.6376	6.8751	0.4528
ZINC13644950	7.0458	7.1921	0.1463	6.9349	0.1109	7.0957	0.0499	7.5311	0.4853	7.1040	0.0582	7.1323	0.0865
ZINC14977337	6.6990	7.3435	0.6445	7.9321	1.2331	7.8929	1.1939	7.7423	1.0433	8.1346	1.4356	8.1566	1.4576
ZINC13644877	6.4685	6.4375	0.0310	5.8472	0.6213	6.1472	0.3213	6.4809	0.0124	5.9945	0.4740	6.1784	0.2901
ZINC13644849	5.6576	5.2325	0.4251	5.3332	0.3244	5.3721	0.2855	5.2872	0.3704	5.1993	0.4583	5.3521	0.3055

* The falling out results are marked by red.

Table S12. Prediction of the pIC_{50} values for the TS3 compounds using models M7–M9.*

Name in ZINC	pIC_{50}^{obs}	M7		M8		M9	
		pIC_{50}^{pred}	$\lvert\Delta pIC_{50}\rvert$	pIC_{50}^{pred}	$\lvert\Delta pIC_{50}\rvert$	pIC_{50}^{pred}	$\lvert\Delta pIC_{50}\rvert$
ZINC29397093	9.0315	8.7366	0.2949	8.9233	0.1082	8.8332	0.1983
ZINC29393614	8.7696	8.5429	0.2267	8.7711	0.0015	8.7343	0.0353
ZINC29396065	7.9393	8.3497	0.4104	8.1527	0.2134	8.0781	0.1388
ZINC29403154	6.6990	7.0591	0.3601	7.4791	0.7801	7.3438	0.6448
ZINC17838260	5.0969	4.5329	0.5640	4.3222	0.7747	4.4594	0.6375
ZINC13726320	4.3979	4.7034	0.3055	4.5319	0.1340	4.6931	0.2952
ZINC13644950	6.2596	6.3845	0.1249	5.8682	0.3914	6.1210	0.1386
ZINC22933690	6.0000	5.6951	0.3049	5.7270	0.2730	5.7743	0.2257
ZINC13644880	5.6778	5.7183	0.0405	5.7040	0.0262	5.7204	0.0426
ZINC13644849	5.3565	5.2764	0.0801	5.1659	0.1906	5.2866	0.0699
ZINC13644854	4.8239	5.5140	0.6901	5.5018	0.6779	5.5360	0.7121
ZINC06119296	4.2218	4.6903	0.4685	5.2189	0.9971	4.9559	0.7341

* The falling out results are marked by red.

Table S13. Prediction of the pIC_{50} values for the TS4 compounds using models M10–M12.*

Name in ZINC	pIC_{50}^{obs}	M10		M11		M12	
		pIC_{50}^{pred}	$\lvert\Delta pIC_{50}\rvert$	pIC_{50}^{pred}	$\lvert\Delta pIC_{50}\rvert$	pIC_{50}^{pred}	$\lvert\Delta pIC_{50}\rvert$
ZINC29402006	9.9586	10.1072	0.1486	9.5460	0.4126	9.4603	0.4983
ZINC29397210	9.7212	9.4090	0.3122	9.6585	0.0627	9.5536	0.1676
ZINC29401607	8.9208	8.3955	0.5253	7.9962	0.9246	8.1263	0.7945
ZINC29399967	7.5229	7.1610	0.3619	7.2828	0.2401	7.3365	0.1864
ZINC29403158	7.0458	8.0392	0.9934	7.3859	0.3401	7.7288	0.6830
ZINC05542666	5.2596	5.2679	0.0083	5.2047	0.0549	5.3261	0.0665
ZINC13602961	4.5229	5.8453	1.3224	5.6892	1.1663	5.8063	1.2834
ZINC26170064	5.0000	4.8313	0.1687	5.0689	0.0689	4.8458	0.1542
ZINC13644953	6.6576	7.2986	0.6410	7.1640	0.5064	7.3539	0.6963
ZINC13644873	6.4202	6.1643	0.2559	6.2660	0.1542	6.2835	0.1367
ZINC13644928	6.1487	6.5627	0.4140	6.3344	0.1857	6.4067	0.2580
ZINC14977328	5.6990	5.8544	0.1554	5.2659	0.4331	5.7526	0.0536

* The falling out results are marked by red.

Table S14. Potential effective thymidine kinase of human herpes viruses HSV-1 and HSV-2 inhibitors selected from the ChEMBL database using virtual screening with QSAR model M3 and M6.

Name in ChEBIL	IC_{50pred}		Selectivity $S = \frac{IC_{50_{hv1}}}{IC_{50_{hv2}}}$
	hv1	hv2	
CHEMBL100259	1775.01	3238.17	0.5482
CHEMBL103283	1074.98	2422.14	0.4438
CHEMBL105318	544.13	1050.03	0.5182
CHEMBL1092065	1775.01	3238.17	0.5482
CHEMBL1199108	15.29	2.87	5.3359
CHEMBL1199070	32.52	13.98	2.3267
CHEMBL1199059	27.75	21.38	1.298
CHEMBL1162943	363.75	1134.23	0.3207
CHEMBL20028	35.85	27.3	1.3131
CHEMBL1231802	849.18	2049.75	0.4143
CHEMBL129	323.89	687.07	0.4714
CHEMBL142873	688.49	1375.31	0.5006
CHEMBL147455	131.74	144.38	0.9125
CHEMBL149078	874.18	1351.76	0.6467
CHEMBL149413	222.59	302.48	0.7359
CHEMBL150007	901.16	1188.78	0.7581
CHEMBL150030	279.51	282.68	0.9888
CHEMBL151287	184.25	256.27	0.719
CHEMBL151398	144.05	79.89	1.8031
CHEMBL151583	205.12	339.86	0.6035
CHEMBL158680	62.73	82.43	0.761
CHEMBL159051	66.87	28.31	2.3617
CHEMBL159063	80.56	154.49	0.5215
CHEMBL1669260	517.13	209.85	2.4643
CHEMBL1669261	527.96	183.02	2.8847
CHEMBL172881	97.07	109.09	0.8898
CHEMBL173904	109.7	144.34	0.76
CHEMBL174063	125.29	140.31	0.8929
CHEMBL174352	224.65	281.32	0.7986
CHEMBL176478	87.56	145.98	0.5998
CHEMBL1780207	30.42	21.46	1.4176
CHEMBL1178256	31.91	5.91	5.4029
CHEMBL19326	14.87	3.82	3.8897
CHEMBL1178302	13.77	3.27	4.2105

CHEMBL19510	9.73	1.37	7.0878
CHEMBL1178307	13.97	2.63	5.321
CHEMBL1956635	429.83	927.26	0.4636
CHEMBL19608	6.88	0.83	8.3308
CHEMBL19725	10.33	2.06	5.0177
CHEMBL19782	8.01	1.41	5.6706
CHEMBL198168	300.89	326.06	0.9228
CHEMBL1178314	8.42	1.52	5.5286
CHEMBL1178315	9.27	1.73	5.3491
CHEMBL20130	275.49	373.85	0.7369
CHEMBL2051768	152.55	178.61	0.8541
CHEMBL2092814	125.29	504.2	0.2485
CHEMBL2093063	125.29	504.2	0.2485
CHEMBL214082	476.54	669.42	0.7119
CHEMBL214700	363.75	1012.98	0.3591
CHEMBL214866	132.59	301.79	0.4393
CHEMBL217675	62.83	26.96	2.3306
CHEMBL221928	651.63	1637.95	0.3978
CHEMBL222030	119.9	207.06	0.5791
CHEMBL222034	312.46	977.46	0.3197
CHEMBL222280	894.75	2094.11	0.4273
CHEMBL223205	149.69	366.1	0.4089
CHEMBL2334552	201.09	344.27	0.5841
CHEMBL236343	237.19	874.78	0.2711
CHEMBL236552	717.13	1631.92	0.4394
CHEMBL2368643	1110.45	1720.68	0.6454
CHEMBL2368647	1226.03	1185.22	1.0344
CHEMBL2368653	268.78	232.27	1.1572
CHEMBL238635	36.62	42.98	0.852
CHEMBL23970	1473.67	3484.98	0.4229
CHEMBL240124	152.09	211.45	0.7193
CHEMBL2403290	26.44	40.28	0.6564
CHEMBL241407	14.48	22.16	0.6535
CHEMBL241408	10.05	6.47	1.5544
CHEMBL244975	652.38	1487.99	0.4384
CHEMBL255062	2449.63	2623.01	0.9339
CHEMBL273695	80.41	135.18	0.5949
CHEMBL274522	219.13	267.55	0.819
CHEMBL1183020	58.72	9.59	6.1247
CHEMBL277025	12.04	1.51	7.9804

CHEMBL277069	128.47	146.05	0.8796
CHEMBL1183046	11.01	0.99	11.094
CHEMBL1183059	43.2	11.15	3.876
CHEMBL277844	5.76	0.7	8.2058
CHEMBL1183063	12.58	2.05	6.1317
CHEMBL1183071	36.69	7.4	4.9562
CHEMBL1183075	15.18	3.11	4.8817
CHEMBL278626	8.87	0.89	9.9477
CHEMBL1183081	12.39	2.53	4.902
CHEMBL1183082	34.36	7.19	4.777
CHEMBL1183089	10.95	1.2	9.1477
CHEMBL1183095	8.78	0.71	12.4135
CHEMBL1183096	7.31	1.04	7.0456
CHEMBL279892	8.78	0.74	11.7868
CHEMBL1183107	14.5	4.72	3.0716
CHEMBL1183108	13.71	3.16	4.3415
CHEMBL280909	5.38	1.07	5.0082
CHEMBL1183123	8.2	0.83	9.8336
CHEMBL1183154	15.3	4.26	3.5958
CHEMBL1183178	11.23	2.77	4.053
CHEMBL1183185	8.32	1.57	5.2872
CHEMBL314011	63.72	12.01	5.3061
CHEMBL3142424	340.25	870.76	0.3907
CHEMBL3142425	119.76	387.44	0.3091
CHEMBL3142561	1073.49	2037.04	0.527
CHEMBL3143653	172.27	733.84	0.2348
CHEMBL3143655	211.69	623.3	0.3396
CHEMBL3143656	117.22	433.81	0.2702
CHEMBL3143666	385.04	1142.62	0.337
CHEMBL3143725	80	155.92	0.5131
CHEMBL3143729	129.66	140.64	0.9219
CHEMBL3143734	166.04	176.93	0.9385
CHEMBL3143735	67.8	121.12	0.5598
CHEMBL3143778	723.44	1409.94	0.5131
CHEMBL3143779	377.83	714	0.5292
CHEMBL3143780	132.86	327.57	0.4056
CHEMBL31634	228.98	756.48	0.3027
CHEMBL318153	1124.6	2437.81	0.4613
CHEMBL3288181	47.18	7.08	6.6674
CHEMBL334676	396.55	1148.42	0.3453

CHEMBL3392172	112.46	100.86	1.1151
CHEMBL3546996	42.61	16.61	2.5651
CHEMBL348540	144.31	269.9	0.5347
CHEMBL352669	48.44	15.78	3.0698
CHEMBL355959	128.47	146.05	0.8796
CHEMBL357505	150.21	193.51	0.7762
CHEMBL358849	865.17	1310.09	0.6604
CHEMBL3589188	321.14	497.97	0.6449
CHEMBL364710	352.05	402.53	0.8746
CHEMBL368940	47.15	69.28	0.6806
CHEMBL372524	56.17	57.13	0.9831
CHEMBL376876	1759.14	2789.97	0.6305
CHEMBL377582	56.17	57.13	0.9831
CHEMBL379296	670.19	1677.64	0.3995
CHEMBL392957	138.77	128.23	1.0822
CHEMBL400410	190.68	98.72	1.9315
CHEMBL400411	243.45	347.54	0.7005
CHEMBL400618	40.82	29.09	1.4034
CHEMBL1185346	8.28	1.06	7.7791
CHEMBL1185463	32.63	6.28	5.1918
CHEMBL1185716	8.81	0.93	9.4314
CHEMBL443308	103.28	286.75	0.3602
CHEMBL477022	93.41	208.07	0.4489
CHEMBL477632	120.28	388.87	0.3093
CHEMBL505732	1037.53	1980.61	0.5238
CHEMBL512059	397.01	1424.3	0.2787
CHEMBL52916	128.47	146.05	0.8796
CHEMBL603916	78.2	39.47	1.9811
CHEMBL604072	1393.8	1784.02	0.7813
CHEMBL605255	223.62	800.94	0.2792
CHEMBL609657	229.3	848.2	0.2703
CHEMBL611519	707.29	1206.7	0.5861
CHEMBL70046	223.62	800.94	0.2792
CHEMBL788	756.14	1183.59	0.6389
CHEMBL83583	80.7	154.14	0.5236
CHEMBL85790	47.64	98.24	0.4849
CHEMBL86280	106.76	22.62	4.7196
CHEMBL917	1166.27	1565.67	0.7449
CHEMBL9579	186.42	277.78	0.6711
CHEMBL9613	75.84	81.13	0.9348

CHEMBL991	894.95	2541.56	0.3521
